# Intratympanic gentamicin for Ménière’s disease

**DOI:** 10.1002/14651858.CD015246.pub2

**Published:** 2023-02-27

**Authors:** Katie E Webster, Kevin Galbraith, Ambrose Lee, Natasha A Harrington-Benton, Owen Judd, Diego Kaski, Otto R Maarsingh, Samuel MacKeith, Jaydip Ray, Vincent A Van Vugt, Martin J Burton

**Affiliations:** Cochrane ENTNuffield Department of Surgical Sciences, University of OxfordOxfordUK; Department of Otolaryngology - Head and Neck SurgeryUniversity of TorontoTorontoCanada; Ménière’s SocietyWootonUK; ENT DepartmentUniversity Hospitals Of Derby and Burton NHS Foundation TrustDerbyUK; National Hospital for Neurology and NeurosurgeryLondonUK; Department of General Practice, Amsterdam UMCVrije Universiteit Amsterdam, Amsterdam Public Health Research InstituteAmsterdamNetherlands; ENT DepartmentOxford University Hospitals NHS Foundation TrustOxfordUK; University of SheffieldSheffieldUK; Cochrane UKOxfordUK

**Keywords:** Adult, Humans, Aminoglycosides, Anti-Bacterial Agents, Anti-Bacterial Agents/adverse effects, Gentamicins, Gentamicins/adverse effects, Meniere Disease, Meniere Disease/complications, Meniere Disease/drug therapy, Tinnitus, Vertigo, Vertigo/drug therapy, Vertigo/etiology

## Abstract

**Background:**

Ménière's disease is a condition that causes recurrent episodes of vertigo, associated with hearing loss and tinnitus. Aminoglycosides are sometimes administered directly into the middle ear to treat this condition. The aim of this treatment is to partially or completely destroy the balance function of the affected ear. The efficacy of this intervention in preventing vertigo attacks, and their associated symptoms, is currently unclear.

**Objectives:**

To evaluate the benefits and harms of intratympanic aminoglycosides versus placebo or no treatment in people with Ménière's disease.

**Search methods:**

The Cochrane ENT Information Specialist searched the Cochrane ENT Register; Central Register of Controlled Trials (CENTRAL); Ovid MEDLINE; Ovid Embase; Web of Science; ClinicalTrials.gov; ICTRP and additional sources for published and unpublished trials. The date of the search was 14 September 2022.

**Selection criteria:**

We included randomised controlled trials (RCTs) and quasi‐RCTs in adults with a diagnosis of Ménière's disease comparing intratympanic aminoglycosides with either placebo or no treatment. We excluded studies with follow‐up of less than three months, or with a cross‐over design (unless data from the first phase of the study could be identified).

**Data collection and analysis:**

We used standard Cochrane methods. Our primary outcomes were: 1) improvement in vertigo (assessed as a dichotomous outcome ‐ improved or not improved), 2) change in vertigo (assessed as a continuous outcome, with a score on a numerical scale) and 3) serious adverse events. Our secondary outcomes were: 4) disease‐specific health‐related quality of life, 5) change in hearing, 6) change in tinnitus and 7) other adverse effects. We considered outcomes reported at three time points: 3 to < 6 months, 6 to ≤ 12 months and > 12 months. We used GRADE to assess the certainty of evidence for each outcome.

**Main results:**

We included five RCTs with a total of 137 participants. All studies compared the use of gentamicin to either placebo or no treatment. Due to the very small numbers of participants in these trials, and concerns over the conduct and reporting of some studies, we considered all the evidence in this review to be very low‐certainty.

**Improvement in vertigo**

This outcome was assessed by only two studies, and they used different time periods for reporting. Improvement in vertigo was reported by more participants who received gentamicin at both 6 to ≤ 12 months (16/16 participants who received gentamicin, compared to 0/16 participants with no intervention; risk ratio (RR) 33.00, 95% confidence interval (CI) 2.15 to 507; 1 study; 32 participants; very low‐certainty evidence) and at > 12 months follow‐up (12/12 participants receiving gentamicin, compared to 6/10 participants receiving placebo; RR 1.63, 95% CI 0.98 to 2.69; 1 study; 22 participants; very low‐certainty evidence). However, we were unable to conduct any meta‐analysis for this outcome, the certainty of the evidence was very low and we cannot draw any meaningful conclusions from the results.

**Change in vertigo**

Again, two studies assessed this outcome, but used different methods of measuring vertigo and assessed the outcome at different time points. We were therefore unable to carry out any meta‐analysis or draw any meaningful conclusions from the results. Global scores of vertigo were lower for those who received gentamicin at both 6 to ≤ 12 months (mean difference (MD) ‐1 point, 95% CI ‐1.68 to ‐0.32; 1 study; 26 participants; very low‐certainty evidence; four‐point scale; minimally clinically important difference presumed to be one point) and at > 12 months (MD ‐1.8 points, 95% CI ‐2.49 to ‐1.11; 1 study; 26 participants; very low‐certainty evidence). Vertigo frequency was also lower at > 12 months for those who received gentamicin (0 attacks per year in participants receiving gentamicin compared to 11 attacks per year for those receiving placebo; 1 study; 22 participants; very low‐certainty evidence).

**Serious adverse events**

None of the included studies provided information on the total number of participants who experienced a serious adverse event. It is unclear whether this is because no adverse events occurred, or because they were not assessed or reported.

**Authors' conclusions:**

The evidence for the use of intratympanic gentamicin in the treatment of Ménière's disease is very uncertain. This is primarily due to the fact that there are few published RCTs in this area, and all the studies we identified enrolled a very small number of participants. As the studies assessed different outcomes, using different methods, and reported at different time points, we were not able to pool the results to obtain more reliable estimates of the efficacy of this treatment. More people may report an improvement in vertigo following gentamicin treatment, and scores of vertigo symptoms may also improve. However, the limitations of the evidence mean that we cannot be sure of these effects. Although there is the potential for intratympanic gentamicin to cause harm (for example, hearing loss) we did not find any information about the risks of treatment in this review.

Consensus on the appropriate outcomes to measure in studies of Ménière's disease is needed (i.e. a core outcome set) in order to guide future studies in this area and enable meta‐analysis of the results. This must include appropriate consideration of the potential harms of treatment, as well as the benefits.

## Plain language summary

**What are the benefits and risks of gentamicin given directly to the ear in Ménière's disease?**

**Key messages**

Due to a lack of robust evidence, it is not clear whether gentamicin ‐ an antibiotic given into the ear (intratympanic gentamicin) works to improve symptoms for people with Ménière's disease. It is also not clear whether there are any risks associated with treatment.

Larger, well‐conducted studies are needed to identify whether this treatment may be effective, and to assess whether there are any harmful effects.

Further work also needs to be done to find out how best to measure the symptoms of people with Ménière's disease, to assess whether treatments are beneficial or not. This should include the development of a 'core outcome set' ‐ a list of things that should be measured in all studies on Ménière's disease.

**What is Ménière's disease?**

Ménière's disease is a condition that affects the inner ear. It causes repeated attacks of dizziness or vertigo (a spinning sensation), together with hearing problems, tinnitus (ringing, humming or buzzing noises in the ear) and a feeling of fullness or pressure in the affected ear. It usually affects adults and starts in middle age.

**How is Ménière's disease treated?**

Oral medications (tablets) are often used as the first treatment for Ménière's disease. If these treatments do not control the symptoms, then a type of antibiotics known as aminoglycosides may be given directly into the ear. Typically this involves an antibiotic called gentamicin. This is most commonly given as an injection through the ear drum.

**What did we want to find out?**

We wanted to find out:

‐ whether there was evidence that intratympanic aminoglycosides (including gentamicin) work at reducing the symptoms of Ménière's disease;

‐ whether this treatment might cause any harm.

**What did we do?**

We searched for studies that compared any intratympanic aminoglycosides to either no treatment or sham (placebo) treatment.

**What did we find?**

We found five studies, which included a total of 137 people. They lasted between six months and two years. All of the studies looked at the antibiotic gentamicin.

‐ More people who received intratympanic gentamicin felt that their vertigo had improved, when compared to people who received no treatment (or a sham treatment). However, the studies were extremely small, so we cannot be sure that the treatment works to improve symptoms.

‐ When people used a scoring system to rate their vertigo symptoms or the frequency of their vertigo, the ratings were better in those who received intratympanic gentamicin when compared to those who received no treatment (or sham treatment). Again, the studies were so small that we are very uncertain if the treatment is effective.

‐ The studies included in this review did not provide any information on the risk of serious harms related to the treatment. Other types of studies have shown that this treatment might be associated with potential side effects (such as complete loss of hearing). However, we do not have any information from the studies included in this review on how common these problems are after treatment.

**What are the limitations of the evidence?**

We have very little confidence in the evidence because most of the studies conducted were very small, and had problems in their conduct, which mean that the results may be unreliable. Larger, well‐conducted studies are needed to try and work out how effective the different treatments really are.

**How up‐to‐date is this evidence?**

This evidence is up‐to‐date to September 2022.

## Summary of findings

**Summary of findings 1:** Intratympanic gentamicin compared to no treatment/placebo for Ménière’s disease

**Intratympanic gentamicin compared to no treatment/placebo for Ménière’s disease**						
**Patient or population:** Ménière’s disease **Setting: **outpatient **Intervention:** intratympanic gentamicin **Comparison:** no treatment/placebo						
**Outcomes**	Anticipated absolute effects^*^ (95% CI)		**Relative effect (95% CI)**	**№ of participants (studies)**	**Certainty of the evidence (GRADE)**	**Comments**
	**Risk with no treatment/placebo**	**Risk with intratympanic gentamicin**				
Improvement in vertigo frequency Assessed with: AAO‐HNS class A, B or C Follow‐up: range 6 months to ≤ 12 months	Actual study population		RR 33.00 (2.15 to 507.00)	32 (1 RCT)	⊕⊝⊝⊝ **very low**^1,2^	Intratympanic gentamicin may increase the number of people who report improvement in the frequency of vertigo at 6 to ≤ 12 months, but the evidence is very uncertain.
	0/16 participants reported that their vertigo had improved	16/16 participants reported that their vertigo had improved				
Improvement in vertigo frequency Assessed with: participants reporting "no vertigo", "significant reduction" or "some reduction" Follow‐up: range > 12 months	Study population		RR 1.63 (0.98 to 2.69)	22 (1 RCT)	⊕⊝⊝⊝ **very low**^2,3,4^	Intratympanic gentamicin may increase the number of people who report improvement in the frequency of vertigo at > 12 months, but the evidence is very uncertain.
	600 participants per 1000 would report that their vertigo had improved	978 participants per 1000 would report that their vertigo had improved (from 588 to 1000)				
Vertigo (global score) Scale from: 0 (none) to 3 (severe) Follow‐up: range 6 months to ≤ 12 months	The mean vertigo score was 1.73 points	MD 1 point lower (1.68 lower to 0.32 lower)	—	26 (1 RCT)	⊕⊝⊝⊝ **very low**^2,4,5,6^	The evidence is very uncertain about the effect of intratympanic gentamicin on global scores of vertigo at 6 to ≤ 12 months.
Vertigo (global score) Scale from: 0 (none) to 3 (severe) Follow‐up: range > 12 months	The mean vertigo score was 1.8 points	MD 1.8 points lower (2.49 lower to 1.11 lower)	—	26 (1 RCT)	⊕⊝⊝⊝ **very low**^2,4,5,6^	Intratympanic gentamicin may reduce global scores of vertigo at > 12 months, but the evidence is very uncertain.
Vertigo (frequency) Assessed with: number of vertigo attacks per year Follow‐up: range > 12 months	The mean number of vertigo attacks per year was 11	The mean number of vertigo attacks per year was 0 (mean difference and 95% CI not estimable)	—	22 (1 RCT)	⊕⊝⊝⊝ **very low**^2,3,4,7^	Intratympanic gentamicin may reduce the frequency of vertigo episodes at > 12 months, but the evidence is very uncertain.
Serious adverse events	No studies reported on this outcome					No information is available on the occurrence of serious adverse events.
*The risk in the intervention group (and its 95% confidence interval) is based on the assumed risk in the comparison group and the **relative effect** of the intervention (and its 95% CI). **AAO‐HNS:** American Academy of Otolaryngology ‐ Head & Neck Surgery; **CI:** confidence interval; **MD:** mean difference; **RCT:** randomised controlled trial; **RR:** risk ratio						
**GRADE Working Group grades of evidence** **High certainty:** we are very confident that the true effect lies close to that of the estimate of the effect. **Moderate certainty:** we are moderately confident in the effect estimate: the true effect is likely to be close to the estimate of the effect, but there is a possibility that it is substantially different. **Low certainty:** our confidence in the effect estimate is limited: the true effect may be substantially different from the estimate of the effect. **Very low certainty:** we have very little confidence in the effect estimate: the true effect is likely to be substantially different from the estimate of effect.						

^1^High risk of bias in 5 out of 7 domains. Quasi‐randomised, unblinded study. Unclear how participants recorded the number of vertigo episodes. ^2^Sample size fails to meet optimal information size, taken as < 300 events for a dichotomous outcome and < 400 participants for a continuous outcome. ^3^Risk of selective reporting, and lack or clarity on how vertigo frequency was assessed in each group. ^4^Extremely small sample size. ^5^High risk of detection bias. Unclear risk of selection and performance bias. ^6^Unvalidated scale used to assess vertigo. ^7^Unable to provide accurate estimate of effect size.

## Background

### Description of the condition

Ménière's disease was first described by Prosper Ménière in 1861 as a condition characterised by episodes of vertigo, associated with hearing loss and tinnitus (Baloh 2001). Sufferers may also report a feeling of fullness in the affected ear. Typically, it initially affects one ear, although some individuals may progress to develop bilateral disease. A hallmark of the condition is that symptoms are intermittent ‐ occurring as discrete attacks that last from minutes to several hours, then resolve. However, over time there is usually a gradual deterioration in hearing, and there may be progressive loss of balance function, leading to chronic dizziness or vertigo.

The diagnosis of Ménière's disease is challenging, due to the episodic nature of the condition, clinical heterogeneity, and the lack of a 'gold standard' diagnostic test. Even the agreed, international classification system has scope for two categories of diagnosis – 'definite' and 'probable' (Lopez‐Escamez 2015). In brief, a diagnosis of definite Ménière's disease requires at least two episodes of vertigo, each lasting 20 minutes to 12 hours, together with audiometrically confirmed hearing loss and fluctuating aural symptoms (reduction in hearing, tinnitus or fullness) in the affected ear. 'Probable' Ménière's disease includes similar features, but without the requirement for audiometry to diagnose hearing loss, and with scope for the vertigo episodes to last longer (up to 24 hours). Both categories ('definite' and 'probable') require that the symptoms are not more likely to be due to an alternative diagnosis, due to the recognised challenges in distinguishing between balance disorders. 

Given the difficulties in diagnosis, the true incidence and prevalence of the disease are difficult to ascertain. A population‐based study in the UK using general practice data estimated the incidence to be 13.1 per 100,000 person‐years (Bruderer 2017), and the prevalence of the disease has been estimated at 190 per 100,000 people in the US (Harris 2010). It is a disorder of mid‐life, with diagnosis typically occurring between the ages of 30 and 60 (Harcourt 2014). Some studies report a slight female preponderance, and there may be a familial association, with approximately 10% of patients reporting the presence of the disease in a first, second or third degree relative (Requena 2014).

The underlying cause of Ménière's disease is usually unknown. Ménière's disease has been associated with an increase in the volume of fluid in the inner ear (endolymphatic hydrops). This may be caused by the abnormal production or resorption of endolymph (Hallpike 1938; Yamakawa 1938). However, it is not clear whether this is the underlying cause of the condition, or merely associated with the disease. Some authors have proposed other underlying causes for Ménière's disease, including viral infections (Gacek 2009), allergic (Banks 2012) or autoimmune disease processes (Greco 2012). A genetic predisposition has also been noted (Chiarella 2015). Occasionally, the symptoms may be secondary to a known cause (such as a head injury or other inner ear disorder) – in these cases it may be referred to as Ménière's syndrome.

Although Ménière's disease is relatively uncommon, it has a profound impact on quality of life. The unpredictable, episodic nature of the condition and severe, disabling attacks of vertigo cause a huge amount of distress. Quality of life (including physical and psychosocial aspects) is significantly reduced for those with Ménière's disease (Söderman 2002). The costs of the condition are also considerable, both in relation to medical interventions (appointments, diagnostic tests and treatments) and loss of productivity or sick days for those affected by the condition (Tyrrell 2016).

### Description of the intervention

A variety of different interventions have been proposed to treat people with Ménière's disease. These include dietary or lifestyle changes, oral treatments, treatments administered by injection into the ear (intratympanic) and surgical treatments. This review focuses on the use of intratympanic aminoglycosides to treat the symptoms of Ménière's disease.

Aminoglycosides are administered into the middle ear via the tympanic membrane. The medication may be administered directly via an injection, or through a tympanostomy tube. The dose and frequency of administration can vary considerably. A number of different regimens have been used, ranging from three doses a day for several consecutive days, to one or two doses spaced a month apart (Chia 2004). Different aminoglycosides have been used, including gentamicin and streptomycin. 

At present, there is no agreement on which is the ideal treatment for people with Ménière's disease – consequently there is no 'gold standard' treatment with which to compare these medications. 

### How the intervention might work

It has long been recognised that aminoglycoside antibiotics carry side effects of problems with balance function and hearing (Hinshaw 1946). These effects are thought to occur through damage and destruction of the hair cells of the inner ear (reviewed in Huth 2011). This mechanism is used therapeutically in Ménière's disease to destroy the balance function of the inner ear. The brain is then able to compensate for the resulting unilateral vestibular hypofunction (assuming function is adequate on the contralateral side). 

The aim of treatment is to destroy (partially or completely) the balance function of the affected ear, whilst preserving hearing. Whilst all aminoglycosides affect the inner ear, gentamicin and streptomycin appear to have predominantly vestibulotoxic (rather than cochleotoxic) effects, and have therefore been used more frequently in Ménière's disease (Selimoglu 2007). However, in some susceptible individuals, the ototoxic effect of aminoglycosides can cause a severe and profound hearing loss. Those with mitochondrial DNA and rRNA mutations are particularly at risk (Murch 2012). 

High‐dose treatment is often given with the aim of eradicating balance function completely. However, some consider that this is not necessary to improve the balance symptoms of Ménière's disease, and that these regimens may increase the risk of damage to hearing (Blakley 2009; Chia 2004). Therefore, many have advocated the use of lower‐dose regimens, or titrating the dose according to the individual response to treatment (Basura 2020). 

### Why it is important to do this review

Balance disorders can be difficult to diagnose and treat. There are few specific diagnostic tests, a variety of related disorders with similar symptoms, and a limited number of interventions that are known to be effective. To determine which topics within this area should be addressed with new or updated systematic reviews we conducted a scoping and prioritisation process, involving stakeholders (https://ent.cochrane.org/balance-disorders-ent). Ménière's disease was ranked as one of the highest priority topics during this process (along with vestibular migraine and persistent postural perceptual dizziness).

Although Ménière's disease is a relatively uncommon condition, the significant impact it has on quality of life demonstrates the clear importance of identifying effective interventions to alleviate the symptoms. There is considerable variation in the management of Ménière's disease on both a national and international scale, with a lack of consensus about appropriate first‐line and subsequent therapies. 

This review is part of a suite of six that consider different interventions for Ménière's disease. Through these reviews, we hope to provide a thorough summary of the efficacy (benefits and harms) of the different treatment options, to support people with Ménière's disease (and healthcare professionals) when making decisions about their care. 

## Objectives

To evaluate the benefits and harms of intratympanic aminoglycosides versus placebo or no treatment in people with Ménière's disease.

## Methods

### Criteria for considering studies for this review

#### Types of studies

We included randomised controlled trials (RCTs) and quasi‐randomised trials (where trials were designed as RCTs, but the sequence generation for allocation of treatment used methods such as alternate allocation, birth dates etc). 

Ménière's disease is known to fluctuate over time, which may mean that cross‐over trials are not an appropriate study design for this condition. No cross‐over RCTs or cluster‐RCTs were identified as relevant for inclusion in this review.

We included studies reported as full‐text, those published as conference abstracts only and unpublished data. 

Ménière's disease is characterised by episodic balance disturbance ‐ the frequency of attacks may change over time (Huppert 2010). For studies to obtain accurate estimates of the effect of different interventions, we considered that follow‐up of participants should be for at least three months ‐ to ensure that participants are likely to have experienced a number of attacks during the follow‐up period. Studies that followed up participants for less than three months were excluded from the review.

#### Types of participants

We included studies that recruited adult participants (aged 18 years or older) with a diagnosis of definite or probable Ménière's disease, according to the agreed criteria of the American Academy Otolaryngology ‐ Head and Neck Surgery (AAO‐HNS), the Japan Society for Equilibrium Research, the European Academy of Otology and Neurotology and the Bárány Society. These criteria are outlined in Appendix 1 and described in Lopez‐Escamez 2015. 

If studies used different criteria to diagnose Ménière's disease, we included them if those criteria were clearly analogous to those described in Lopez‐Escamez 2015. For example, studies that used earlier definitions of Ménière's disease (from the AAO‐HNS guidelines of 1995) were also included. If there was uncertainty over the criteria used for the study, then a decision was made on whether to include the study. This decision was taken by authors who were masked to other features of the studies (such as study size, other aspects of methodology, results of the study) to avoid the introduction of bias in study selection. If a study was conducted in an ENT department and participants were diagnosed with Ménière's disease then we considered it was likely that other diagnoses had been excluded, and included the study. However, we reflected this uncertainty in diagnosis by considering the study at risk of indirectness when using GRADE to assess the certainty of the evidence (see 'Summary of findings and assessment of certainty of the evidence). 

We anticipated that most studies would include participants with active Ménière's disease. We did not exclude studies if the frequency of attacks at baseline was not reported or was unclear, but we planned to highlight if there were differences between studies that may impact on our ability to pool the data, or affect the applicability of our findings.

We excluded studies where participants had previously undergone destructive/ablative treatment for Ménière's disease in the affected ear (such as vestibular neurectomy, chemical or surgical labyrinthectomy), as we considered that they were unlikely to respond to interventions in the same way as those who had not undergone such treatment.

#### Types of interventions

We included the following interventions:

- gentamicin.

If we had identified other intratympanic aminoglycosides (for example, streptomycin) then these would also have been included in the review, but we did not find any relevant studies that considered other aminoglycosides. 

The main comparison is:

- gentamicin versus placebo/no treatment.

##### Concurrent treatments

There were no limits on the type of concurrent treatments used, providing these were used equally in each arm of the study. We pooled studies that included concurrent treatments with those where participants did not receive concurrent treatment. We planned to conduct subgroup analysis to determine whether the effect estimates may be different in those receiving additional treatment. However, due to the small number of studies included in the review this was not possible (see Subgroup analysis and investigation of heterogeneity).

#### Types of outcome measures

We assessed all outcomes at the following time points: 

- 3 to < 6 months;
- 6 to ≤ 12 months;
- > 12 months.

The exception was for adverse event data, when we used the longest time period of follow‐up. 

We searched the COMET database for existing core outcome sets of relevance to Ménière's disease and vertigo, but were unable to find any published core outcome sets. We therefore conducted a survey of individuals with experience of (or an interest in) balance disorders to help identify the outcomes that should be prioritised. This online survey was conducted with the support of the Ménière's Society and the Migraine Trust, and included 324 participants, who provided information regarding priority outcomes. The review author team used the results of this survey to inform the choice of outcome measures in this review. 

We analysed the following outcomes in the review, but did not use them as a basis for including or excluding studies.

##### Primary outcomes

- Improvement in vertigo
  - Measured as a dichotomous outcome (improved/not improved), according to self‐report, or according to a change of a specified score (as described by the study authors) on a vertigo rating scale.
- Change in vertigo
  - Measured as a continuous outcome, to identify the extent of change in vertigo symptoms.
- Serious adverse events
  - Including any event that causes death, is life‐threatening, requires hospitalisation, results in disability or permanent damage, or in congenital abnormality. Measured as the number of participants who experience at least one serious adverse event during the follow‐up period. We also looked for data on the occurrence of the following specified, serious adverse event:
    - complete loss of hearing in the affected ear.

Vertigo symptoms comprise a variety of different features, including frequency of episodes, duration of episodes and severity/intensity of the episodes. Where possible, we included data for the vertigo outcomes that encompassed all of these three aspects (frequency, duration and severity/intensity of symptoms). However, we anticipated that these data may not be available from all studies. We therefore extracted data on the frequency of vertigo episodes as an alternative measure for these outcomes. 

##### Secondary outcomes

- Disease‐specific health‐related quality of life
  - Measured with the Dizziness Handicap Inventory (DHI, Jacobsen 1990), a validated measurement scale in widespread use. If data from the DHI are unavailable we extracted data from alternative validated measurement scales, according to the order of preference described in the list below (based on the validity of the scales for this outcome):
    - DHI short form (Tesio 1999);
    - DHI screening tool (Jacobsen 1998);
    - Vertigo Handicap Questionnaire (Yardley 1992a);
    - Ménière's Disease Patient Oriented Symptoms Inventory (MD POSI, Murphy 1999);
    - University of California Los Angeles Dizziness Questionnaire (UCLADQ, Honrubia 1996);
    - AAO‐HNS Functional Level Scale (FLS, AAO‐HNS 1995).
- Hearing
  - Measured with pure tone audiometry and reported as the change in pure tone average (PTA), or (alternatively) by patient report, if data from PTA were not available.
- Tinnitus
  - Measured using any validated, patient‐reported questionnaire relating to the impact of tinnitus, for example the Tinnitus Handicap Inventory (THI, Newman 1996) or the Tinnitus Functional Index (TFI, Meikle 2012).
- Other adverse effects
  - Measured as the number of participants who experience at least one episode of the specified adverse events during the follow‐up period. Including the following specified adverse effects:
    - tympanic membrane perforation;
    - ear pain;
    - post‐injection vertigo;
    - new onset tinnitus in the affected ear.

### Search methods for identification of studies

The Cochrane ENT Information Specialist conducted systematic searches for randomised controlled trials and controlled clinical trials in October 2021 and September 2022. There was no language, publication year or publication status restriction. The date of the search was 14 September 2022.

#### Electronic searches

The Information Specialist searched:

- the Cochrane ENT Trials Register (search via the Cochrane Register of Studies to 14 September 2022);
- the Cochrane Central Register of Controlled Trials (CENTRAL) (search via the Cochrane Register of Studies to 14 September 2022);
- Ovid MEDLINE(R) Epub Ahead of Print, In‐Process & Other Non‐Indexed Citations, Ovid MEDLINE(R) Daily and Ovid MEDLINE(R) (1946 to 14 September 2022);
- Ovid Embase (1974 to 14 September 2022);
- Web of Knowledge, Web of Science (1945 to 14 September 2022;
- ClinicalTrials.gov, www.clinicaltrials.gov (to 14 September 2022);
- World Health Organization (WHO) International Clinical Trials Registry Platform (ICTRP), https://trialsearch.who.int/ (to 14 September 2022).

The Information Specialist modelled subject strategies for databases on the search strategy designed for CENTRAL. The strategy has been designed to identify all relevant studies for a suite of reviews on various interventions for Ménière's disease. Where appropriate, they were combined with subject strategy adaptations of the highly sensitive search strategy designed by Cochrane for identifying randomised controlled trials and controlled clinical trials (as described in the *Cochrane Handbook for Systematic Reviews of Interventions* Version 5.1.0, Box 6.4.b, Handbook 2011). Search strategies for major databases including CENTRAL are provided in Appendix 2.

#### Searching other resources

We scanned the reference lists of identified publications for additional trials and contacted trial authors where necessary. In addition, the Information Specialist searched Ovid MEDLINE to retrieve existing systematic reviews relevant to this systematic review, so that we could scan their reference lists for additional trials. In addition, the Information Specialist ran a non‐systematic search of Google Scholar to identify trials not published in mainstream journals. 

We did not perform a separate search for adverse effects. We considered adverse effects described in included studies only.

### Data collection and analysis

#### Selection of studies

The Cochrane ENT Information Specialist used the first two components of Cochrane's Screen4Me workflow to help assess the search results: 

1. Known assessments – a service that matches records in the search results to records that have already been screened in Cochrane Crowd and been labelled as 'a RCT' or as 'not a RCT'. 
2. The machine learning classifier (RCT model) (Wallace 2017), available in the Cochrane Register of Studies (CRS‐Web), which assigns a probability of being a true RCT (from 0 to 100) to each citation. Citations that were assigned a probability score below the cut‐point at a recall of 99% were assumed to be non‐RCTs. We manually dual screened the results for those that scored on or above the cut‐point. 

At least two review authors (including KG, AL, KW) or a co‐worker (BG and SC, listed in Acknowledgements) independently screened the remaining titles and abstracts using Covidence, to identify studies that may be relevant for the review. Any discrepancies were resolved by consensus, or by retrieving the full text of the study for further assessment. 

We obtained the full text for any study that was considered possibly relevant and two authors (including KG, AL, KW) or a co‐worker (BG) again independently checked this to determine whether it met the inclusion criteria for the review. Any differences were resolved by discussion and consensus, or through recourse to a third author if necessary. 

We listed excluded any studies that were retrieved in full text but subsequently deemed to be inappropriate for the review (according to the inclusion/exclusion criteria), according to the main reason for exclusion. 

The unit of interest for the review is the study, therefore multiple papers or reports of a single study are grouped together under a single reference identification. The process for study selection is recorded in Figure 1. 

**1:**
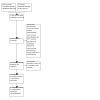
Flow chart of study retrieval and selection.

##### Screening eligible studies for trustworthiness

We assessed studies meeting our inclusion criteria for trustworthiness using a screening tool developed by Cochrane Pregnancy and Childbirth. This tool includes specified criteria to identify studies that are considered sufficiently trustworthy to be included in the review (see Appendix 3 and Figure 2). If studies were assessed as being potentially 'high‐risk', we attempted to contact the study authors to obtain further information or address any concerns. We planned to exclude studies from the main analyses of the review if there were persisting concerns over trustworthiness, or we were unable to contact the authors. However, over the course of the review it became apparent that the majority of included studies had some concerns ‐ typically due to missing information that was not reported in the original study publications. 

**2:**
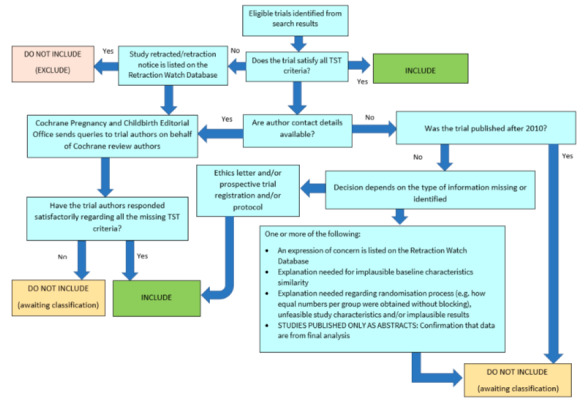
The Cochrane Pregnancy and Childbirth Trustworthiness Screening Tool

Only one of our included studies had no concerns when using the trustworthiness screening tool (Bremer 2014). The remaining studies all presented very limited baseline characteristics for participants, which prevented us from assessing whether randomisation seemed to be adequate. In addition, three studies reported that no participants were lost to follow‐up during the trial (Choudhary 2019; Stokroos 2004; Ul Shamas 2017). Two studies had additional concerns over the process used for randomisation, and we were unable to identify a trial protocol or prospective registration, despite the trial taking place since 2010 (Choudhary 2019; Ul Shamas 2017). 

We attempted to contact study authors to clarify these issues, but we either received no reply, or the authors were unable to access the original trial data to clarify our queries. 

There are several possible explanations for the large number of studies that had concerns when using the tool. One is that there are issues with the trustworthiness of the studies identified in this review, and the data included may not give reliable estimates of the true effect. Alternatively, the trustworthiness screening tool may be excessively sensitive, and flag studies that are trustworthy, but where information has not been fully reported. We note that this tool (and others used for the same purpose) has not yet been validated for use. 

We therefore took the decision to include the studies in the review, despite the potential concerns over trustworthiness. The uncertainty in the results is captured as part of our GRADE rating in the certainty of the evidence, using the domain 'study limitations'. 

#### Data extraction and management

Two review authors (KG, KW) independently extracted outcome data from each study using a standardised data collection form. Where a study had more than one publication, we retrieved all publications to ensure complete extraction of data. Any discrepancies in the data extracted by the two authors were checked against the original reports, and differences were resolved through discussion and consensus. If required, we contacted the study authors for clarification.

We extracted data on the key characteristics of the studies, including the following information:

- study design, duration of the study, number of study centres and location, study setting and dates of the study;
- information on the participants, including the number randomised, those lost to follow‐up or withdrawn, the number analysed, the age of participants, gender, severity of the condition, diagnostic criteria used, inclusion and exclusion criteria for the individual studies;
- details of the intervention, comparator, and concomitant treatments or excluded medications;
- the outcomes specified and reported by the study authors, including the time points;
- funding for the study and any conflicts of interest for the study authors;
- information required to assess the risk of bias in the study, and to enable GRADE assessment of the evidence.

Once the extracted data were checked and any discrepancies resolved, a single author transferred the information to Review Manager 5 (RevMan 2020). 

The primary effect of interest for this review is the effect of treatment assignment (which reflects the outcomes of treatment for people who were assigned to the intervention) rather than a per protocol analysis (the outcomes of treatment only for those who completed the full course of treatment as planned). For the outcomes of interest in this review, we extracted the findings from the studies on an available case basis, i.e. all available data from all participants at each time point, based on the treatment to which they were randomised. This was irrespective of compliance, or whether participants had received the intervention as planned.

In addition to extracting pre‐specified information about study characteristics and aspects of methodology relevant to risk of bias, we extracted the following summary statistics for each study and outcome:

- For continuous data: the mean values, standard deviation and number of patients for each treatment group at the different time points for outcome measurement. Where change‐from‐baseline data were not available, we extracted the values for endpoint data instead. If values for the individual treatment groups were not reported, where possible we extracted summary statistics (e.g. mean difference) from the studies.
- For binary data: we extracted information on the number of participants experiencing an event, and the number of participants assessed at that time point. If values for the individual treatment groups were not reported, where possible we extracted summary statistics (e.g. risk ratio) from the studies.
- For ordinal scale data: if the data appeared to be normally distributed, or if the analysis performed by the investigators indicated that parametric tests are appropriate, then we treated the outcome measure as continuous data. Alternatively, if data were available, we converted these to binary data for analysis ‐ for example, for analysis of improvement in vertigo, when rated using the AAO‐HNS 1995 control of vertigo scale. 
- For time‐to‐event data: we did not identify any time‐to‐event data for the outcomes specified in the review. 

If necessary, we converted data found in the studies to a format appropriate for meta‐analysis, according to the methods described in the *Cochrane Handbook for Systematic Reviews of Interventions* (Handbook 2021). 

We pre‐specified time points of interest for the outcomes in this review. Where studies reported data at multiple time points, we took the longest available follow‐up point within each of the specific time frames. For example, if a study reported an outcome at 12 weeks and 20 weeks of follow‐up then the 20‐week data was included for the time point 3 to 6 months (12 to 24 weeks).

#### Assessment of risk of bias in included studies

Two authors (KG, KW) undertook assessment of the risk of bias of the included studies independently, with the following taken into consideration, as guided by the *Cochrane Handbook for Systematic Reviews of Interventions* (Handbook 2011):

- sequence generation;
- allocation concealment;
- blinding;
- incomplete outcome data;
- selective outcome reporting; and
- other sources of bias.

We used the Cochrane risk of bias tool (Handbook 2011), which involves describing each of these domains as reported in the study and then assigning a judgement about the adequacy of each entry: 'low', 'high' or 'unclear' risk of bias.

#### Measures of treatment effect

We summarised the effects of the majority of dichotomous outcomes (e.g. serious adverse effects) as risk ratios (RR) with 95% confidence intervals (CIs). We have also expressed the results as absolute numbers based on the pooled results and compared to the assumed risk in the summary of findings table (Table 1) and full GRADE profile (Table 2).

**1:** GRADE profile: intratympanic gentamicin for Ménière's disease

**Certainty assessment**							**Number of participants**		**Effect**			
**№ of studies**	**Study design**	**Risk of bias**	**Inconsistency**	**Indirectness**	**Imprecision**	**Other considerations**	**Intratympanic gentamicin**	**No treatment/placebo**	**Relative** **(95% CI)**	**Absolute** **(95% CI)**	** Certainty**	**Comments **
**Improvement in vertigo frequency (follow‐up: range 6 months to ≤ 12 months; assessed with: AAO‐HNS class A, B or C)**												
1	Randomised trial	Very serious^a^	Not serious	Not serious	Serious^b^	None	16/16 (100.0%)	Actual improvement in this study: 0/16 (0.0%)	**RR 33.00** (2.15 to 507.00)	Not estimable	⊕⊝⊝⊝ Very low	
								If the improvement in the control group was 1.0%		**320 more per 1000** (from 12 more to 1000 more)		
								If the improvement in the control group was 10.0%		**1000 more per 1000** (from 115 more to 1000 more)		
**Improvement in vertigo frequency (follow‐up: range > 12 months; assessed with: participants reported "no vertigo", "significant reduction" or "some reduction")**												
1	Randomised trial	Serious^c^	Not serious	Not serious	Very serious^b,d^	None	12/12 (100.0%)	6/10 (60.0%)	**RR 1.63** (0.98 to 2.69)	**378 more per 1000** (from 12 fewer to 1000 more)	⊕⊝⊝⊝ Very low	
**Improvement in vertigo frequency: sensitivity analysis for complete/substantial improvement (follow‐up: range 6 months to ≤ 12 months; assessed with: AAO‐HNS 1995 class A or B)**												
1	Randomised trial	Very serious^a^	Not serious	Not serious	Serious^b^	None	16/16 (100.0%)	Actual improvement in this study: 0/16 (0.0%)	**RR 33.00** (2.15 to 507.00)	Not estimable	⊕⊝⊝⊝ Very low	
								If the improvement in the control group was 1.0%		**320 more per 1000** (from 12 more to 1000 more)		
								If the improvement in the control group was 10.0%		**1000 more per 1000** (from 115 more to 1000 more)		
**Improvement in vertigo frequency: sensitivity analysis for complete/substantial improvement (follow‐up: range > 12 months; assessed with: participant reported "no vertigo" or "significant reduction")**												
1	Randomised trial	Serious^c^	Not serious	Not serious	Very serious^b,d^	None	12/12 (100.0%)	1/10 (10.0%)	**RR 7.05** (1.59 to 31.32)	**605 more per 1000** (from 59 more to 1000 more)	⊕⊝⊝⊝ Very low	
**Change in vertigo (global score) (follow‐up: range 6 months to ≤ 12 months; scale from: 0 (none) to 3 (severe))**												
1	Randomised trial	Serious^e^	Not serious	Serious^f^	Very serious^b,d^	None	16	10	‐	MD **1 point lower** (1.68 lower to 0.32 lower)	⊕⊝⊝⊝ Very low	
**Change in vertigo (global score) (follow‐up: range > 12 months; scale from: 0 (none) to 3 (severe))**												
1	Randomised trial	Serious^e^	Not serious	Serious^f^	Very serious^b,d^	None	16	10	‐	MD **1.8 points lower** (2.49 lower to 1.11 lower)	⊕⊝⊝⊝ Very low	
**Change in vertigo (frequency) (follow‐up: range > 12 months; assessed with: number of vertigo attacks per year)**												
1	Randomised trial	Serious^c^	Not serious	Not serious	Very serious^b,d,g^	None	12 Mean: 0 attacks per year	10 Mean: 11 attacks per year	‐	Not estimable	⊕⊝⊝⊝ Very low	
**Change in hearing (follow‐up: range > 12 months; assessed with: pure tone audiometry)**												
2	Randomised trials	Serious^h^	Serious^i^	Not serious	Serious^b^	None	28	21	‐	MD **3.7 dbHL higher** (8.29 lower to 15.69 higher)	⊕⊝⊝⊝ Very low	

**CI:** confidence interval; **MD:** mean difference; **RR:** risk ratio ^a^High risk of bias in five out of seven domains. Quasi‐randomised, unblinded study. Unclear how participants recorded the number of vertigo episodes. ^b^Sample size fails to meet optimal information size, taken as < 300 events for a dichotomous outcome and < 400 participants for a continuous outcome. ^c^Risk of selective reporting, and lack or clarity on how vertigo frequency was assessed in each group. ^d^Extremely small sample size. ^e^High risk of detection bias. Unclear risk of selection and performance bias. ^f^Unvalidated scale used to assess vertigo. ^g^Unable to provide accurate estimate of effect size. ^h^One study at high risk of detection bias. Both studies at unclear risk of selection bias. ^i^I^2^ = 44%. Effect direction varies between trials, although likely to be a trivial effect.

For continuous outcomes, we expressed treatment effects as a mean difference (MD) with standard deviation (SD). We did not need to use standardised mean difference to pool any data. 

#### Unit of analysis issues

Ménière's disease is unlikely to be a stable condition, and interventions may not have a temporary effect. If cross‐over trials were identified then we planned to use only the data from the first phase of the study. If cluster‐randomised trials were identified then we would have ensured that analysis methods were used to account for clustering in the data (Handbook 2021). However, no cross‐over or cluster‐randomised trials were identified for inclusion. 

We identified two studies with three arms (Bremer 2014; Ul Shamas 2017). The two arms in Bremer 2014 related to the same comparison (high‐dose and low‐dose gentamicin) therefore we included these data by pooling the two intervention arms, to avoid double‐counting of any participants (according to the methods in the Handbook 2021). Only two arms in Ul Shamas 2017 were relevant to this review (gentamicin and placebo) therefore the third arm, intratympanic dexamethasone, was disregarded. These data are included in a companion review on intratympanic corticosteroids for Ménière's disease (Webster 2021a).

#### Dealing with missing data

We planned to contact study authors via email whenever the outcome of interest was not reported, if the methods of the study suggest that the outcome had been measured. We did the same if not all data required for meta‐analysis were reported (for example, standard deviations), unless we were able to calculate them from other data reported by the study authors. 

#### Assessment of heterogeneity

We assessed clinical heterogeneity by examining the included studies for potential differences between them in the types of participants recruited, interventions or controls used and the outcomes measured. This is highlighted in the Included studies section, below.

We used the I^2^ statistic to quantify inconsistency among the studies in each meta‐analysis. We also considered the P value from the Chi^2^ test. However, few meta‐analyses were conducted in the course of this review, and we did not identify any serious inconsistency. 

#### Assessment of reporting biases

We assessed reporting bias as within‐study outcome reporting bias and between‐study publication bias.

##### Outcome reporting bias (within‐study reporting bias)

We assessed within‐study reporting bias by comparing the outcomes reported in the published report against the study protocol or trial registry, whenever this could be obtained. If the protocol or trial registry entry was not available, we compared the outcomes reported to those listed in the methods section. If results are mentioned but not reported adequately in a way that allows analysis (e.g. the report only mentions whether the results were statistically significant or not), bias in a meta‐analysis is likely to occur. We then sought further information from the study authors. If no further information was found, we noted this as being a 'high' risk of bias with the risk of bias tool. If there was insufficient information to judge the risk of bias we noted this as an 'unclear' risk of bias (Handbook 2011). 

##### Publication bias (between‐study reporting bias)

We did not have sufficient studies to create funnel plots for any analysis. We did not identify any ongoing studies that remain unpublished. We are therefore unable to comment on the potential for publication bias in this review. 

#### Data synthesis

##### Meta‐analysis of numerical data

Where possible and appropriate (if participants, interventions, comparisons and outcomes were sufficiently similar in the trials identified) we conducted a quantitative synthesis of results. We conducted all meta‐analyses using RevMan 2020. We anticipated that the underlying effect of the intervention may vary between studies, due to differences between participants, settings and the interventions used for each study. We planned to use a random‐effects model for meta‐analysis and explore whether the use of a fixed‐effect model substantially alters the effect estimates (see Sensitivity analysis). However, we were only able to use the Peto odds ratio (OR) ‐ a fixed‐effect method ‐ for some meta‐analyses in this review, due to rare or zero events in at least one of the studies included in the analysis.

For dichotomous data, we plan to analyse treatment differences as a risk ratio (RR) calculated using the Mantel‐Haenszel methods.

For continuous outcomes, if all data were from the same scale, we pooled mean follow‐up values with change‐from‐baseline data and reported this as a mean difference. We did not need to report standardised mean differences in this review.

Improvement in vertigo symptoms may be assessed using a variety of methods, which consider different aspects of vertigo. These include:

- frequency of vertigo episodes;
- duration of vertigo episodes;
- severity/intensity of vertigo episodes;
- a composite measure of all of these aspects:
  - for example, assessed with a global score ‐ such as "how troublesome are your vertigo symptoms?", rated on an ordinal scale.

For the outcomes "improvement in vertigo" and "change in vertigo", we planned to prioritise outcome measures that use a composite score ‐ encompassing aspects of vertigo frequency, duration and severity/intensity. Examples of this may include a global rating scale of vertigo impact (rated from 0 to 10, where 0 is defined as no symptoms, and 10 is defined as the most troublesome symptoms) or the vertigo/balance subscale of the Vertigo Symptom Scale (Yardley 1992b), or Vertigo Symptom Scale Short Form (Yardley 1998). As data from composite scores were not available from the majority of studies, then we also included data on the frequency of vertigo episodes as an alternative measure.

##### Synthesis using other methods

If we were unable to pool numerical data in a meta‐analysis for one or more outcomes we planned to provide a synthesis of the results using alternative methods, following the guidance in chapter 12 of the Handbook 2021. However, this was not necessary, as results were typically provided by a single study. 

#### Subgroup analysis and investigation of heterogeneity

If statistical heterogeneity was identified for any comparisons, we planned to assess this considering the following subgroups:

- Different doses/frequency of administration.
- Use of concomitant treatment.
- Diagnosis of Ménière's disease.

Regardless of statistical heterogeneity, if sufficient data were available, we planned to explore the impact of baseline hearing on the outcome 'hearing'. As many people receiving intratympanic aminoglycosides would already have very poor hearing, the impact of further hearing loss may not be functionally significant. 

However, due to the paucity of data available, and the few meta‐analyses included in this review, we did not carry out any subgroup analysis. 

#### Sensitivity analysis

We planned to carry out a number of sensitivity analyses for the primary outcomes in this review. However, the paucity of data and the lack of meta‐analyses has meant that this was not possible. 

If few studies are identified for meta‐analysis, the random‐effects model may provide an inaccurate measure of the between‐studies variance. Therefore, we explored the impact of using a fixed‐effect model using a sensitivity analysis, and the results are very similar (Table 3).

**2:** Sensitivity analyses

**Primary analysis**	**Sensitivity analysis result**	**Description of analysis**
Analysis 1.5: Change in hearing at > 12 months	MD 5.28 (95% CI ‐2.57 to 13.13)	Fixed‐effect model

CI: confidence interval; MD: mean difference

If there was uncertainty over the diagnostic criteria used for participants in the studies (for example, if it was not clear whether participants were diagnosed using criteria that are analogous to the AAO‐HNS criteria) then we also planned to explore this by including/excluding those studies from the analysis. However, all the studies included in this review used the AAO‐HNS 1995 criteria to diagnose Ménière's disease. 

We used the Cochrane Pregnancy and Childbirth Screening Tool to identify any studies with concerns over the data available. We had intended that any studies identified by the tool would be excluded from the main analyses in the review, but that we would explore the impact of including the data from these studies through a sensitivity analysis. However, as noted above, we had some concerns over the use of this tool, and few studies were included in the review, therefore this sensitivity analysis was not conducted. 

We did conduct one sensitivity analysis that was not pre‐specified in our protocol. When drafting the protocol for this review we stated "improvement in vertigo" as our outcome. However, over the course of the review it became apparent that "any improvement" may not represent a meaningful improvement for people with Ménière's disease. For example, an individual who suffered 100 vertigo attacks per year at baseline and then only 99 attacks per year at follow‐up could be stated to have 'improved' ‐ although it is not clear whether the difference would be of any importance. 

For our main analysis for this outcome we considered 'any improvement' in vertigo, but we also conducted a sensitivity analysis to see if the effect estimates were altered if we considered 'substantial improvement' in vertigo. 

#### Summary of findings and assessment of the certainty of the evidence

Two independent authors (AL, KW) used the GRADE approach to rate the overall certainty of evidence using GRADEpro GDT (https://gradepro.org/) and the guidance in chapter 14 of the *Cochrane Handbook for Systematic Reviews of Interventions* (Handbook 2021). Disagreements were resolved through discussion and consensus. The certainty of evidence reflects the extent to which we are confident that an estimate of effect is correct, and we have applied this in the interpretation of results. There are four possible ratings: high, moderate, low and very low. A rating of high certainty of evidence implies that we are confident in our estimate of effect and that further research is very unlikely to change our confidence in the estimate of effect. A rating of very low certainty implies that any estimate of effect obtained is very uncertain.

The GRADE approach rates evidence from RCTs that do not have serious limitations as high certainty. However, several factors can lead to the downgrading of the evidence to moderate, low or very low. The degree of downgrading is determined by the seriousness of these factors:

- Study limitations (risk of bias)
  - This was assessed using the rating from the Cochrane risk of bias tool for the study or studies included in the analysis. We rated down either one or two levels, depending on the number of domains which had been rated at high or unclear risk of bias. 
- Inconsistency
  - This was assessed using the I^2^ statistic and the P value for heterogeneity for all meta‐analyses, as well as by visual inspection of the forest plot. For results based on a single study we rated this domain as no serious inconsistency.
- Indirectness of evidence
  - We took into account whether there were concerns over the population included in these study or studies for each outcome, as well as whether additional treatments were offered that may impact on the efficacy of the intervention under consideration. 
- Imprecision
  - We took into account the sample size and the width of the confidence interval for each outcome. If the sample size did not meet the optimal information size (i.e. < 400 people for continuous outcomes or < 300 events for dichotomous outcomes), or the confidence interval crossed the small effect threshold we rated down one level. If the sample size did not meet the optimal information size and the confidence interval included both potential harm and potential benefit we rated down twice. We also rated down twice for very tiny studies (e.g. 10 to 15 participants in each arm), regardless of the estimated confidence interval.
- Publication bias
  - We considered whether there were likely to be unpublished studies that may impact on our confidence in the results obtained. 

We used a minimally contextualised approach, and rated the certainty in the interventions having an important effect (Zeng 2021). Where possible, we used agreed minimally important differences (MIDs) for continuous outcomes as the threshold for an important difference. Where no MID was identified, we provide an assumed MID based on agreement between the authors. For dichotomous outcomes, we looked at the absolute effects when rating imprecision, but also took into consideration the GRADE default approach (rating down when a RR crosses 1.25 or 0.80). We have justified all decisions to downgrade the certainty of the evidence using footnotes, and added comments to aid the interpretation of the findings, where necessary. 

We have provided a summary of findings tables for the only comparison:

- Intratympanic gentamicin versus placebo/no treatment

We have included all primary outcomes in the summary of findings table. We planned to prioritise outcomes at the time point three to six months for presentation in the tables. However, no data were available at these time points for any outcomes, and therefore we have shown the data for longer periods of follow‐up. We have also included a full GRADE profile for all results (see Table 2).

## Results

### Description of studies

#### Results of the search

The searches in September 2022 retrieved a total of 4434 records. This reduced to 3408 after the removal of duplicates. The Cochrane ENT Information Specialist sent all 3408 records to the Screen4Me workflow. The Screen4Me workflow identified 122 records as having previously been assessed: 83 had been rejected as not RCTs and 39 had been assessed as possible RCTs. The RCT classifier rejected an additional 1427 records as not RCTs (with 99% sensitivity). We did not send any records to the Cochrane Crowd for assessment. Following this process, the Screen4Me workflow had rejected 1510  records and identified 1898 possible RCTs for title and abstract screening. 

**Table:** 

** **	**Possible RCTs**	**Rejected**
Known assessments	39	83
RCT classifier	1859	1427
Total	1898	1510

We identified 89 additional duplicates. We screened the titles and abstracts of the remaining 1809 records. We discarded 1791 records and assessed 18 full‐text records.

We excluded 12 records (linked to 11 studies) with reasons recorded in the review (see Excluded studies). 

We included five completed studies (six records) where results were available. 

A flow chart of study retrieval and selection is provided in Figure 1.

#### Included studies

We included a total of five RCTs (Bremer 2014; Choudhary 2019; Postema 2008; Stokroos 2004; Ul Shamas 2017). Details of the individual studies can be found in the Characteristics of included studies table. 

##### Study design

All included studies were described as randomised controlled trials. Three studies were conducted in the Netherlands (Bremer 2014; Postema 2008; Stokroos 2004), and two studies were conducted in India (Choudhary 2019; Ul Shamas 2017). Most of the studies included only two study arms, comparing gentamicin to placebo or no intervention (Choudhary 2019; Postema 2008; Stokroos 2004). Two studies were three‐armed trials: Bremer 2014 compared two different doses of gentamicin to placebo and Ul Shamas 2017 compared gentamicin to both corticosteroids and placebo. The comparison of corticosteroids to placebo from Ul Shamas 2017 is included in a separate review in this suite (Webster 2021a).

The minimum duration of follow‐up was six months (Choudhary 2019; Ul Shamas 2017). Participants were followed for 12 months in one study (Postema 2008), and for between 6 and 28 months in one further study (Stokroos 2004). The authors of Bremer 2014 planned to follow up participants for two years; however, the study was terminated prematurely. It appears that most participants in this study would have been followed up for more than one year. 

##### Participants

All the included studies recruited adult participants with a diagnosis of Ménière's disease. 

###### Diagnosis of Ménière's disease

All included studies reported the use of the AAO‐HNS 1995 criteria to diagnose Ménière's disease. 

###### Features of Ménière's disease

Four studies stated that only those with definite disease were included (Bremer 2014; Choudhary 2019; Postema 2008; Stokroos 2004), and three studies only include participants with unilateral disease (Bremer 2014; Postema 2008; Stokroos 2004).

Only one study provided information about the duration of symptoms. Participants in Bremer 2014 had symptoms for approximately three years before enrolment into the study. Most studies indicated that participants needed to have used some form of medical treatment for their Ménière's disease for at least six months without improvement in their symptoms, before enrolment into the study.

The frequency of vertigo attacks at baseline was only described by one study. Participants in Stokroos 2004 had a mean frequency of 25 vertigo attacks per year in the placebo arm, and 74 attacks per year in the gentamicin arm. This marked discrepancy in baseline attack frequency between the groups may have impacted on the results. 

##### Interventions and comparisons

All the included studies used gentamicin as the active intervention. However, the dose and frequency of administration varied across the studies. 

- Postema 2008 used a 12 mg injection of gentamicin, administered once per week for a total of four weeks (48 mg in total). The drug (and comparator) were administered through a ventilation tube.
- Choudhary 2019 used a 20 mg gentamicin injection. This was repeated up to a total of three times (60 mg in total) depending on the relief of symptoms.
- Stokroos 2004 used a gentamicin solution of 30 mg/mL. The authors stated that 4 mL of solution was drawn up (120 mg), but it is not clear whether this was all instilled ‐ we considered it likely that a maximum of approximately 1 mL would be used (30 mg). The injection could also be repeated once every six weeks, until a maximum of 360 mg gentamicin had been instilled.
- Ul Shamas 2017 used a single injection of 80 mg gentamicin solution.
- Bremer 2014 used a gentamicin solution of 40 mg/mL, but did not state the quantity instilled at each administration. Participants received either two or four injections of active treatment (for low‐dose and high‐dose treatment).

Most studies compared gentamicin to placebo injection(s), except for Choudhary 2019, which compared the intervention to no treatment. 

##### Outcomes

###### 1. Improvement in vertigo

For this outcome we included dichotomous data ‐ assessed as the proportion of participants whose vertigo had 'improved' or 'not improved'. Only two studies assessed this outcome. 

####### 1.1. Global score

No studies reported the improvement of vertigo using a global score that considered the frequency, duration and intensity of vertigo attacks. 

####### 1.2. Frequency

Choudhary 2019 assessed improvement in the frequency of vertigo using the AAO‐HNS 1995 'control of vertigo' scale. The number of vertigo attacks in the interval after treatment is divided by the number of vertigo spells prior to treatment and multiplied by 100. The resulting number indicates the extent of ‘control of vertigo’ or CoV. The AAO‐HNS further divides the control of vertigo into classes, where class A (CoV = 0) represents a complete control of vertigo, class B (CoV 1% to 40%) represents a substantial control of vertigo, class C (41% to 80%) limited control, class D (81% to 120%) insignificant control and class E (> 120%) worse control (deterioration). When assessing any improvement in vertigo, we considered participants with a CoV of A, B or C to have experienced improvement, and those with a CoV of D or E to have not improved. For the sensitivity analysis of substantial improvement or complete resolution of vertigo we considered participants with a CoV of A or B to have substantial improvement/complete resolution and those with CoV C, D or E to have not had this degree of improvement. 

Stokroos 2004 assessed improvement in vertigo frequency using a four‐point scale: 'no complaints', 'significant reduction', 'some reduction' or 'no benefit'. We did not identify any validation of this rating scale. For the main analysis we included participants with any reduction in vertigo, i.e. either no complaints, a significant reduction or some reduction. For the sensitivity analysis we included those with 'no complaints' or a 'significant reduction' in vertigo frequency. 

###### 2. Change in vertigo

This outcome included data on the change in vertigo using a continuous numerical scale. Data were reported by two studies.  

####### 2.1. Global score

A single study assessed the change in vertigo using a global score (Postema 2008). Participants self‐rated their vertigo as 'severe', 'moderate', 'mild' or 'none'. We thought that it was likely they would have considered the severity, frequency and duration of vertigo episodes when selecting a rating, but this is not explicit in the article. 

####### 2.2. Frequency

Stokroos 2004 reported on the number of vertigo attacks per year at baseline and at the end of follow‐up. 

###### 3. Serious adverse events

This outcome included any event that caused death, was life‐threatening, required hospitalisation, resulted in disability or permanent damage, or in congenital abnormality. Serious adverse events were stated to be assessed by Bremer 2014 but were not actually reported ‐ it is unclear whether this was because no events occurred. The remaining studies did not describe any serious adverse events but, again, it is unclear whether this was because adverse events were not assessed, not reported, or did not occur.

###### 4. Disease‐specific health‐related quality of life

Only Bremer 2014 reported on this outcome, and used the Dizziness Handicap Inventory (DHI) to assess quality of life. 

###### 5. Hearing

Pure tone audiometry (PTA) was used to assess hearing status in three studies, using the average hearing threshold at 0.5 kHz, 1 kHz, 2 kHz and 4 kHz (Bremer 2014; Postema 2008; Stokroos 2004). However, no variance was reported by Bremer 2014, therefore the results could not be included in our meta‐analysis. The studies Choudhary 2019 and Ul Shamas 2017 provided very little information on how hearing was assessed and analysed, therefore the results could not be included in our analysis. 

###### 6. Tinnitus

Three studies did not assess tinnitus (Bremer 2014; Choudhary 2019; Stokroos 2004). Postema 2008 did not use a validated scale to assess the impact of tinnitus on quality of life (as stated in our protocol), therefore the data are not included for this outcome (tinnitus was self‐rated as severe, moderate, mild or none). Ul Shamas 2017 stated that the THI 'grade' was used to assess tinnitus, but there are only limited details on how this outcome was assessed, therefore we could not include the data in this review. 

###### 7. Other adverse effects

None of the included studies reported on the other adverse effects of treatment that were of interest in this review (tympanic membrane perforation, ear pain, post‐injection vertigo and new onset tinnitus in the affected ear).

#### Excluded studies

After assessing the full text, we excluded 11 studies from this review. The main reason for exclusion for each study is listed below. 

Four studies were not randomised controlled trials (Graybiel 1967; Guo 2016; Nedzelski 1992; Thabet 2008).

We identified a number of review articles that did not provide any primary outcome data. This included two narrative reviews (Conde 1965; Richards 1971), and five systematic reviews or meta‐analyses (Diamond 2003; Dimitriadis 2017; Hao 2022; Huon 2012; Syed 2015). We checked the reference lists of the systematic reviews and meta‐analyses, to ensure that we had already identified any relevant studies. 

### Risk of bias in included studies

See Figure 3 for the risk of bias graph (our judgements about each risk of bias item presented as percentages across all included studies) and Figure 4 for the risk of bias summary (our judgements about each risk of bias item for each included study). All the studies included had some concerns regarding the risk of bias, with at least one domain being rated at unclear or high risk of bias. 

**3:**
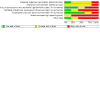
Risk of bias graph (our judgements about each risk of bias item presented as percentages across all included studies.

**4:**
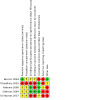
Risk of bias summary (our judgements about each risk of bias item for each included study).

#### Allocation

Only one study provided sufficient information on the generation of a random sequence for us to be confident that an adequate method of randomisation was used (Bremer 2014). The methods used for randomisation in one study were inadequate (Choudhary 2019), and the remaining three studies did not provide information on the methods used for randomisation, therefore we judged them at unclear risk of bias. 

The methods used to conceal allocation were unclear in most studies, except for Choudhary 2019, where there was clearly a risk of bias from a lack of allocation concealment. 

#### Blinding

Two studies were open‐label trials, where study participants, personnel and outcome assessors were all aware of the group allocation (Choudhary 2019; Ul Shamas 2017). We judged these at high risk of both performance and detection bias. 

Two studies indicated that study participants and personnel were blinded to group allocation (Postema 2008; Stokroos 2004). However, Postema 2008 reported that blinding was 'broken' during the final visit, which we considered may be a risk for detection bias. 

The final study did indicate that the study design included blinding for participants, study personnel and outcome assessors (Bremer 2014). However, the authors explicitly state that participants were advised to visit a physiotherapist after intratympanic gentamicin injections, for Cawthorne‐Cooksey exercises. It is not clear whether this intervention was also offered to those who received placebo injections. Consequently, we rated this as an unclear risk of performance and detection bias. 

#### Incomplete outcome data

The number of participants who dropped out of Bremer 2014 was not balanced across the intervention groups, which has the potential to cause some bias in the results. One further study did not provide any information on attrition, therefore we rated it at unclear risk of bias (Ul Shamas 2017). We considered the remaining three studies to be at low risk of attrition bias, as they reported complete follow‐up, or the extent of dropout was not considered to be sufficient to cause bias in the results. 

#### Selective reporting

We rated the majority of the included studies at high risk of selective reporting bias. The study Bremer 2014 only reported on some of the pre‐specified outcome measures listed in the trial protocol. This may have been because of the premature termination of the trial. Choudhary 2019 reported some outcome information incompletely, or for only one of the intervention groups. This prevented us from conducting a comparison of the efficacy between groups, and may lead to bias in the analysis. One study did not assess any outcomes relating to vertigo, which we considered to be unusual for a trial of treatments for Ménière's disease (Ul Shamas 2017). The study Stokroos 2004 also had a discrepancy in the outcome reporting between the results section and the discussion section of the article. Although the main results section indicated that no patients in the gentamicin group had any vertiginous episodes at follow‐up, the discussion section indicates that one participant did develop a recurrence of their symptoms. 

Finally, we considered the study Postema 2008 to be at unclear risk of selective reporting, as we were unable to identify a published trial protocol for this study. 

#### Other potential sources of bias

We rated one study at unclear risk of other bias, as very limited information was presented regarding the methods and conduct of the study (Choudhary 2019). We rated the studies Postema 2008 and Stokroos 2004 at high risk for this domain, due to the use of unvalidated scoring systems to assess vertigo, and a lack of clarity on how participants assessed their symptoms. We also rated the study Ul Shamas 2017 at high risk of other bias, as very limited details were provided on the study methods, and data were not reported in a way that allowed adequate comparison of the intervention and control groups.

### Effects of interventions

See: Table 1

#### 1. Intratympanic gentamicin compared to no treatment/placebo

##### 1.1. Improvement in vertigo

For this outcome we included dichotomous data ‐ assessed as the proportion of participants whose vertigo had 'improved' or 'not improved'. 

###### 1.1.1. Improvement in global score

No studies measured global improvement in vertigo ‐ taking account of the frequency, severity or intensity and duration of symptoms. 

###### 1.1.2. Improvement in frequency

Two studies assessed improvement in the frequency of vertigo.

####### 1.1.2.1. At 3 to < 6 months

No data were reported at this time point. 

####### 1.1.2.2. At 6 to ≤ 12 months

 Choudhary 2019 reported at this time point. The risk ratio (RR) for any improvement was 33.00 in those receiving intratympanic gentamicin (16/16 participants receiving gentamicin, compared to 0/16 participants receiving no treatment; 95% confidence interval (CI) 2.15 to 570.00; 1 study; 32 participants; very low‐certainty evidence; Analysis 1.1).

####### 1.1.2.3. At > 12 months

Stokroos 2004 reported at this time point. The risk ratio for any improvement was 1.63 in those receiving intratympanic gentamicin (12/12 participants receiving gentamicin, compared to 6/10 participants receiving placebo; 95% CI 0.98 to 2.69; 1 study; 22 participants; very low‐certainty evidence; Analysis 1.1).

Our protocol stated that this primary outcome measure should be any "improvement" in vertigo, therefore in the analyses above we have included data that consider participants who had any degree of improvement. However, we note that class C vertigo control includes a reduction in the frequency of episodes of between 20% and 59%. We considered that a reduction of only 20%, or "some reduction" in vertigo, may not be viewed as an important change in the frequency of episodes by many people with Ménière's disease, or by healthcare professionals. Indeed, a number of publications considered only class A or B as 'improvement'. Therefore, we explored whether assessing those with complete or substantial control of vertigo would change our effect estimates. At 6 to ≤ 12 months the results were identical (95% CI 2.15 to 570.00; 1 study; 32 participants; very low‐certainty evidence; Analysis 1.2). At > 12 months the RR for improvement was 7.05 (12/12 participants receiving gentamicin, compared to 1/10 participants receiving no treatment; 95% CI 1.59 to 31.32; 1 study; 22 participants; very low‐certainty evidence; Analysis 1.2). Although the certainty of the evidence is very low throughout, this may indicate that a stronger effect is seen when considering only complete or substantial improvement in vertigo, rather than any improvement. 

##### 1.2. Change in vertigo

This outcome included data on the change in vertigo using a continuous numerical scale. 

###### 1.2.1. Change in global score

A single study reported on the change in vertigo using a global score, which included the frequency of episodes, the severity or intensity of symptoms and the duration of episodes (Postema 2008). This study used a four‐point scale for participants to report their vertigo symptoms, which ranged from 0 (no vertigo) to 3 (severe vertigo).  

####### 1.2.1.1. At 3 to < 6 months

No data were reported at this time point. 

####### 1.2.1.2. At 6 to ≤ 12 months

The mean difference in vertigo score was ‐1.00 in those who received gentamicin, indicating lower (better) scores in this group (95% CI ‐1.68 to ‐0.32; 1 study; 26 participants; very low‐certainty evidence; Analysis 1.3).

####### 1.2.1.3. At > 12 months

The mean difference in vertigo score was ‐1.80 in those who had received gentamicin (95% CI ‐2.49 to ‐1.11; 1 study; 26 participants; very low‐certainty evidence; Analysis 1.3). 

###### 1.2.2. Change in frequency

Stokroos 2004 reported on the number of vertigo attacks over the course of a year. This was the only study to report on the change in vertigo frequency. 

####### 1.2.2.1. At 3 to < 6 months

No data were reported at this time point. 

####### 1.2.2.2. At 6 to ≤ 12 months

No data were reported at this time point. 

####### 1.2.2.3. At > 12 months

All 12 participants who received gentamicin reported no episodes of vertigo over the course of a year. In contrast, those who received placebo had a mean of 11 attacks per year (with a standard deviation (SD) of 10). No confidence interval could be calculated (due to the SD of zero in the intervention group), but the mean difference between the groups would therefore be a reduction of 11 episodes per year, in favour of the intervention group (1 study; 22 participants; very low‐certainty evidence; Analysis 1.4).

##### 1.3. Serious adverse events

Very limited information was available on serious adverse events. None of the included studies reported on the total number of participants who suffered severe adverse events. For most of the studies it is unclear whether this was because no serious adverse events occurred, or because the data were not collected (Choudhary 2019; Postema 2008; Stokroos 2004; Ul Shamas 2017). The study Bremer 2014 specifically states that "Harms were reported following the CONSORT extension for harms", and methods for this are outlined in the protocol. However, the total number of serious adverse events is not reported, although one participant in the low‐dose gentamicin group died of a comorbidity. 

We specifically looked for data on the number of participants who developed complete hearing loss over the course of the study, however no studies fully reported this. Again, it is not clear whether this is because no participants developed hearing loss, or because this adverse event was not specifically assessed and reported. The study Ul Shamas 2017 states that two participants developed "profound sensorineural hearing loss", but it is not clear which treatment group these participants were allocated to (intratympanic gentamicin, intratympanic corticosteroids or placebo). We attempted to contact the author to clarify this, but received no reply.  

##### 1.4. Disease‐specific health‐related quality of life

A single study assessed this outcome (Bremer 2014). The Dizziness Handicap Inventory (DHI) was used to measure this outcome, however only median scores and ranges are reported. The authors report that the high‐dose gentamicin group "showed the largest decrease in DHI score but this was not statistically significant (P>0.5)". 

##### 1.5. Change in hearing

All included studies assessed hearing in some way. Postema 2008 and Stokroos 2004 both assessed hearing loss with the extended Fletcher index (the mean of hearing thresholds at 0.5 kHz, 1 kHz, 2 kHz and 4 kHz).

Choudhary 2019 provided some information on the 'progression of hearing loss', reporting that this was experienced by 2/16 (12.5%) participants who received gentamicin, compared to 0/16 (0%) of participants who received no treatment. However, there is no information on how this was assessed, and what reduction in hearing threshold was considered 'progression'. Ul Shamas 2017 reported some information on the number of participants who experienced deterioration of hearing (16), and the number who developed profound sensorineural hearing loss (2). However, it is not clear which of the treatment groups these participants were allocated to (intratympanic corticosteroids, intratympanic gentamicin or placebo). 

###### 1.5.1. At 3 to < 6 months

No data were reported at this time point. 

###### 1.5.2. At 6 to ≤ 12 months

No data were reported at this time point. 

###### 1.5.3. At > 12 months

Overall, the mean change in hearing level for the gentamicin group increased (worsened) by 3.70 dB (95% CI ‐8.29 to 15.69; 2 studies; I^2^ = 44%; very low‐certainty evidence; Analysis 1.5). However, it should be noted that there was some heterogeneity in this analysis. The direction of effect varied between the two studies. However, the effect size may represent a trivial difference between the groups.  

Bremer 2014 also reported on the change in hearing level, but only provided the mean change in the three groups, without a measure of the standard deviation, therefore these data could not be included in the meta‐analysis. The change was reported as an increase of 0.9 dB for the low‐dose gentamicin group, an increase of 27.4 dB for the high‐dose gentamicin group and an increase of 10 dB in the placebo group.

##### 1.6. Change in tinnitus

No studies reported on this outcome measure using a validated scale that considered the impact of tinnitus on quality of life. 

##### 1.7. Other adverse effects

No studies reported on the other adverse effects of relevance to this review (tympanic membrane perforation, ear pain, post‐injection vertigo or new onset tinnitus in the affected ear).

## Discussion

### Summary of main results

The five studies included in this review all considered the use of intratympanic gentamicin. The evidence identified was all of very low certainty, therefore we have very low confidence in the estimates of effects. 

Intratympanic gentamicin may increase the proportion of people who experience an improvement in their vertigo symptoms, and the proportion who experience a substantial improvement in their vertigo symptoms, at both 6 to ≤ 12 months and > 12 months, but the evidence is very uncertain. Similarly, global ratings of vertigo may be better in those who receive intratympanic gentamicin at 6 to ≤ 12 months and > 12 months, and the frequency of attacks may be lower at > 12 months. However, all of this evidence is also of very low certainty. 

Two studies reported on the change in hearing threshold, and found little to no difference between those who received intratympanic gentamicin and those who received placebo. Again, the evidence is of very low certainty. 

We did not identify any information on the risk of serious adverse effects, disease‐specific health‐related quality of life, tinnitus or other adverse effects. 

### Overall completeness and applicability of evidence

Despite intratympanic aminoglycosides being in common use for refractory Ménière's disease, there is a lack of evidence from randomised controlled trials on the efficacy and harms of this intervention. We identified only five studies, all of which compared intratympanic gentamicin to placebo or no treatment, enrolling a total of just 137 participants. 

We did not identify any information on serious (or less‐serious) adverse effects of treatment. This is surprising for an intervention that has recognised effects on hearing (Selimoglu 2007). There is currently insufficient evidence for us to judge whether the use of aminoglycosides (at the dose and frequency used in these studies) has a significant impact on hearing. Most of the studies included in this review used intratympanic injections for administration of gentamicin. This procedure may itself carry a risk of adverse effects ‐ such as ear discharge or tympanic membrane perforation ‐ regardless of the material injected. Therefore when balancing the risks and benefits of this procedure, individuals with Ménière's disease may wish to have information on the frequency with which these events occur as a consequence of intratympanic injection. However, we did not find any information on this risk from the studies included in this review.  

It is noteworthy that ‐ in this situation ‐ evidence regarding the risks of an intervention may come from different types of studies to those which consider efficacy. Clearly, placebo interventions are required to appropriately consider the efficacy of an intervention such as intratympanic gentamicin. However, when the procedure itself (intratympanic injection) is associated with specific risks, it is also relevant to compare the intervention to no treatment ‐ in order to appropriately gather information on the absolute risk of harms. 

We are aware of a number of studies that compare intratympanic gentamicin to intratympanic corticosteroids ‐ these are not included in this review. The symptoms of Ménière's disease often fluctuate over time ‐ and may improve or worsen regardless of any treatment. Therefore, to establish whether intratympanic aminoglycosides are genuinely effective, we considered that they should be compared directly with no treatment or placebo. 

This review was conducted as part of a suite considering different interventions for Ménière's disease. A number of issues were identified as affecting the completeness and applicability of the evidence in all the reviews in this suite. These have been described in the companion review on systemic pharmacological interventions for Ménière's disease (Webster 2021b) and are replicated here, as they relate to this review:

- There is a paucity of evidence about all of these interventions, despite some of them being in common use for Ménière’s disease. All the evidence we found was of very low or low certainty, showing that we are unsure of the effects of the interventions, and future research may change the effect estimates a great deal.
- We were unable to carry out many meta‐analyses. Although we identified five studies for inclusion, there were often differences in the actual outcomes assessed in the study, or the time points for follow‐up. Therefore, we were unable to pool the data to achieve a more precise estimate of any effect. Finally, study authors often used different ways of measuring the same outcome, which prevented data from being combined. For example, vertigo was assessed with either a global score, or a frequency score, which could not be combined, or hearing was assessed using a continuous scale or as an improvement above a certain threshold. 
- Certain outcomes were only assessed by some included studies. Many studies did not assess the impact of the disease on quality of life or tinnitus at all. Potential adverse effects of the interventions were also often poorly reported or simply not assessed.
- We noted that unvalidated rating scales were commonly used in the studies included, particularly when looking at the global impact of treatments for vertigo. When such scales are used, it is difficult to know if they are accurately assessing the outcome, and also what size of change on this scale represents a meaningful difference in the outcome (the minimally important difference). 
- Finally, studies often failed to report clearly what treatments participants received before joining the trial, what maintenance treatment they continued on during the trial, and whether they received any additional treatments over the course of the trial. The impact of these additional treatments may be considerable, particularly for those studies with longer‐term follow‐up. Without knowing the background details of study participants (for example, the duration of their Ménière's disease, or what treatments they have tried in the past) it is difficult to identify the groups of people who may benefit from these treatments. 

### Quality of the evidence

We used the GRADE approach to assess the certainty of the evidence in this review. The evidence identified was all low‐ or very low‐certainty, meaning that we are uncertain about the actual effect of these interventions for all of our outcomes. The main issues that affected the certainty of the evidence were the domains of study limitations and imprecision. The different domains addressed by GRADE are considered in more detail below.

#### Study limitations/risk of bias

All the studies included in this review had concerns regarding the potential for bias in the study design, conduct or reporting. Most studies did not provide a clear description of methods used to randomise participants into groups, or to conceal allocation, therefore we rated these domains at unclear risk of bias. However, we acknowledge that this may be in part due to poor reporting, rather than the actual conduct of the studies. One study used a quasi‐randomised method, resulting in a high risk of selection bias and bias by confounding (Choudhary 2019). 

Two studies did not appear to mask participants, study personnel or outcome assessors to the interventions used in each group, leading to a high or unclear risk of performance and detection bias (Choudhary 2019; Ul Shamas 2017). One study was at risk of attrition bias due to differential dropout between the groups (Bremer 2014). We had substantial concerns about the risk of selective reporting in this review. We rated four trials at high risk for this domain, due to incomplete reporting of outcomes that had been pre‐specified in the trial protocol/registration (Bremer 2014), incomplete reporting of results that precluded their inclusion in this review (Choudhary 2019; Ul Shamas 2017), and differences in outcome reporting in the results and discussion sections (Stokroos 2004). We had additional concerns about the conduct of three studies, leading to a high risk of 'other bias' ‐ predominantly due to concerns over methods used for assessing and reporting outcomes (Postema 2008; Stokroos 2004; Ul Shamas 2017).

#### Inconsistency

Few meta‐analyses were conducted in the course of this review, therefore inconsistency did not usually impact on the certainty of the evidence. For the majority of outcomes, a single study was included in the analysis. Consequently, inconsistency between studies was not of relevance. We only had one meta‐analysis where inconsistency was considered to be a concern (Analysis 1.5).

#### Indirectness

This was not a major concern for most of the outcomes. We rated down for indirectness if there was significant concern over the methods used to measure an outcome (for example, use of an unvalidated scoring system for vertigo, as in Postema 2008).

#### Imprecision

Many included studies were small and, as discussed above, we were unable to carry out meta‐analyses. Therefore, the total sample size for each of our outcomes of interest was small, and reduced the certainty of the evidence. For some outcomes the resulting confidence intervals for the effect size were also extremely wide ‐ meaning that there was uncertainty over whether the intervention was beneficial or harmful. This further impacted on the certainty of the evidence. 

For each analysis result, the width of the confidence interval is compared to the threshold for an important difference (details of how these thresholds were selected are described in the Methods section). If the confidence interval crosses this threshold ‐ and includes both the potential for an important benefit and the potential for a trivial effect, then the certainty of the evidence would be reduced by one level. If the confidence interval includes the possibility of *both* an important benefit and an important harm then the certainty would be reduced further. Therefore, it is important to agree on thresholds for this rating, i.e. where is the threshold, or cut‐point, between a trivial difference and a small, but important benefit or harm for each outcome? This question is difficult to answer, and requires input from people with balance disorders. As part of this review process, one of the author team (KW) joined some discussion groups for people with balance disorders, to try and obtain their views on quantifying an important and meaningful difference in treatment outcomes. However, the main theme that emerged from these discussions was that people were unable to give a specific threshold for each outcome. Instead, individuals tended to weigh up a variety of different factors when determining this threshold. The invasiveness and burden of taking the treatment would be taken into account, as well as potential side effects and the severity of their symptoms at that time. The GRADE working group would likely refer to this as a "fully contextualised approach", accounting for all aspects of the specific intervention in order to set thresholds for benefit (Zeng 2021). For this review we adopted a "minimally contextualised approach" and rated imprecision for each outcome according to specific, defined thresholds (as described in Methods). However, if the thresholds used are inappropriate then this may affect the certainty of the evidence (by a maximum of one level). 

#### Other considerations

We did not rate down the certainty of the evidence for other reasons. Publication bias is usually assessed as part of this domain. Although we are aware that this is an issue with many systematic reviews, we did not find strong indications of publication bias with this review.

### Potential biases in the review process

We made some small changes to the review process following the publication of our protocol (Webster 2021c). 

Firstly, we planned to use the Cochrane Pregnancy and Childbirth Trustworthiness Tool to assess the included studies. We had planned to exclude any study where there were concerns (as identified with this tool) from the main analyses. However, as described above, we were unable to determine whether most of the included studies would pass the screening tool, either due to a lack of reporting in the original articles, or because we were unable to contact the authors to resolve any issues. If these studies were subsequently found to have genuine concerns over research integrity then this would further undermine our confidence in the findings of the review. However, as the evidence for these interventions is almost all very low‐certainty, we considered that this would not greatly impact the findings of the review. 

We also identified that the outcome "improvement in vertigo" may not capture an important change in vertigo. Therefore, we added a sensitivity analysis for this outcome. For our main analysis we considered any improvement in vertigo, as pre‐planned. However, we also looked at whether considering "complete resolution of vertigo, or a substantial improvement in vertigo", would impact on the effect estimates. We did note that the point estimate showed a larger effect size at > 12 months when using this analysis (results were identical at 6 to ≤ 12 months), but the evidence remained very low‐certainty, therefore we cannot draw any firm conclusions from this exploratory approach. 

### Agreements and disagreements with other studies or reviews

A previous Cochrane Review on this topic included two of the five studies we identified in this review (Pullens 2011). The authors of this review concluded that intratympanic gentamicin seems to be an effective treatment for vertigo in Ménière's disease, but may carry a risk of hearing loss. Although our review includes some of the same data, our findings are different ‐ predominantly because we have used the GRADE approach to express our certainty in the effects seen. 

One further systematic review and meta‐analysis on this topic also concluded that intratympanic gentamicin may be an effective treatment for Ménière's disease (Zhang 2019). Most of the studies included in this review were 'before and after' cohort studies. The authors concluded that intratympanic gentamicin may result in good control of vertigo, but highlighted the need for large, high‐quality RCTs in this area. Three older reviews based on cohort studies also concluded that there was some evidence for the efficacy of intratympanic gentamicin (Chia 2004; Cohen‐Kerem 2004; Diamond 2003).

## Authors' conclusions

**Implications for practice**

The evidence for the use of intratympanic gentamicin for Ménière's disease is very uncertain. This is predominantly due to the small size of randomised controlled trials (RCTs) in this area, and some methodological concerns with the conduct and reporting of these studies. 

**Implications for research**

This review was conducted as part of a suite regarding different interventions for Ménière's disease. Many of the conclusions below are relevant to all of these reviews and are replicated across the suite.

The lack of high‐certainty RCT evidence for intratympanic aminoglycosides suggests that well‐conducted studies with larger numbers of participants are required to appropriately assess the efficacy (and potential harms) of this intervention. However, there also needs to be more clarity on which outcomes studies should assess, and when and how to assess them. Vertigo is a notoriously difficult symptom to assess, and there is great variety in the methods used to record and report this symptom in the studies we have identified. 

There is a clear need for consensus on which outcomes are important to people with Ménière’s disease, so that future studies can be designed with this in mind. Development of a core outcome set would be preferable as a guide for future trials. We understand that development of a core outcome set for Ménière's disease was underway, with a project registered on the COMET website (https://www.comet-initiative.org/Studies/Details/818), but we have been unable to identify any results of this project, or ascertain whether it is ongoing. If a core outcome set is developed, this should include details on the recommended methods used to measure outcomes, ensuring that these are validated, reliable tools. Monitoring and reporting of adverse effects should be considered a routine part of any study, and should always occur ‐ this is inconsistent at present. Agreement is also needed on the appropriate times at which outcomes should be measured to adequately assess the different interventions.

Any decisions about which outcomes to measure, how to measure them and when to measure them must be made with input from people with Ménière’s disease, to ensure that the outcomes reported by trialists (and future systematic reviews) are relevant to those with the disease. 

For those considering development of a core outcome set, we would highlight that the use of a dichotomous outcome (such as 'improvement' or 'no improvement') may be challenging. Ideally, agreement should be reached on what constitutes a *meaningful change* in symptoms when using this method to categorise outcomes. This is relevant for both vertigo outcomes (where there may be differences in the number of people who experience *any* improvement, compared to the number who experience a *substantial* improvement) and also hearing outcomes ‐ where a slight deterioration in hearing may be tolerable, but substantial hearing loss may not.  

Trialists should also be clear about the treatments that participants received before entry to the trial, throughout the trial, and the need for additional treatment during the course of the trial. People with Ménière's disease need to be able to understand whether interventions work in all people with the disease, or whether they might work best during certain phases of the disease ‐ perhaps as a first‐line therapy, or for people in whom other treatments have failed. 

## History

Protocol first published: Issue 12, 2021

## Appendices

### Appendix 1. AAO‐HNS definition of Ménière's disease

Definite Ménière's disease:

- Two or more spontaneous episodes of vertigo, each lasting 20 minutes to 12 hours.
- Audiometrically documented low‐ to medium‐frequency sensorineural hearing loss in one ear, defining the affected ear on at least one occasion before, during or after one of the episodes of vertigo.
- Fluctuating aural symptoms (hearing, tinnitus or fullness) in the affected ear.
- Not better accounted for by another vestibular diagnosis.

Probable Ménière's disease: 

- Two or more spontaneous episodes of vertigo, each lasting 20 minutes to 24 hours.
- Fluctuating aural symptoms (hearing, tinnitus or fullness) in the affected ear.
- Not better accounted for by another vestibular diagnosis.

Taken from Lopez‐Escamez 2015.

### Appendix 2. Search strategies

This search strategy has been designed to identify all relevant studies for a suite of reviews on various interventions for Ménière's disease. 

**Table:**

**CENTRAL (CRS) **	**Cochrane ENT Register (CRS) **	**MEDLINE (Ovid) **
1 MESH DESCRIPTOR Endolymphatic Hydrops EXPLODE ALL AND CENTRAL:TARGET 2 meniere*):AB,EH,KW,KY,MC,MH,TI,TO AND CENTRAL:TARGET 3 (endolymphatic near hydrops):AB,EH,KW,KY,MC,MH,TI,TO AND CENTRAL:TARGET 4 (labyrinth* near hydrops):AB,EH,KW,KY,MC,MH,TI,TO AND CENTRAL:TARGET 5 (labyrinth* near syndrome):AB,EH,KW,KY,MC,MH,TI,TO AND CENTRAL:TARGET 6 (aural near vertigo):AB,EH,KW,KY,MC,MH,TI,TO AND CENTRAL:TARGET 7 (labyrinth* near vertigo):AB,EH,KW,KY,MC,MH,TI,TO AND CENTRAL:TARGET 8 (cochlea near hydrops):AB,EH,KW,KY,MC,MH,TI,TO AND CENTRAL:TARGET 9 (vestibular near hydrops):AB,EH,KW,KY,MC,MH,TI,TO AND CENTRAL:TARGET 10 #1 OR #2 OR #3 OR #4 OR #5 OR #6 OR #7 OR #8 OR #9 AND CENTRAL:TARGET	1 MESH DESCRIPTOR Endolymphatic Hydrops EXPLODE ALL AND INREGISTER 2 (meniere*):AB,EH,KW,KY,MC,MH,TI,TO AND INREGISTER 3 (endolymphatic near hydrops):AB,EH,KW,KY,MC,MH,TI,TO AND INREGISTER 4 (labyrinth* near hydrops):AB,EH,KW,KY,MC,MH,TI,TO AND INREGISTER 5 (labyrinth* near syndrome):AB,EH,KW,KY,MC,MH,TI,TO AND INREGISTER 6 (aural near vertigo):AB,EH,KW,KY,MC,MH,TI,TO AND INREGISTER 7 (labyrinth* near vertigo):AB,EH,KW,KY,MC,MH,TI,TO AND INREGISTER 8 (cochlea near hydrops):AB,EH,KW,KY,MC,MH,TI,TO AND INREGISTER 9 (vestibular near hydrops):AB,EH,KW,KY,MC,MH,TI,TO AND INREGISTER 10 #1 OR #2 OR #3 OR #4 OR #5 OR #6 OR #7 OR #8 OR #9 AND INREGISTER 11 INREGISTER 12 * AND CENTRAL:TARGET 13 #11 NOT #12 14 #10 AND #13	1 exp Endolymphatic Hydrops/ 2 meniere*.ab,ti. 3 (endolymphatic adj3 hydrops).ab,ti. 4 (labyrinth* adj3 hydrops).ab,ti. 5 (labyrinth* adj3 syndrome).ab,ti. 6 (aural adj3 vertigo).ab,ti. 7 (labyrinth* adj3 vertigo).ab,ti. 8 (cochlea adj3 hydrops).ab,ti. 9 (vestibular adj3 hydrops).ab,ti. 10 1 or 2 or 3 or 4 or 5 or 6 or 7 or 8 or 9 11 randomized controlled trial.pt. 12 controlled clinical trial.pt. 13 randomized.ab. 14 placebo.ab. 15 drug therapy.fs. 16 randomly.ab. 17 trial.ab. 18 groups.ab. 19 11 or 12 or 13 or 14 or 15 or 16 or 17 or 18 20 exp animals/ not humans.sh. 21 19 not 20 22 10 and 21
**Embase (Ovid) **	**Web of Science Core Collection (Web of Knowledge) **	**Trial Registries **
1 exp Meniere disease/ 2 meniere*.ab,ti. 3 (endolymphatic adj3 hydrops).ab,ti. 4 (labyrinth* adj3 hydrops).ab,ti. 5 (labyrinth* adj3 syndrome).ab,ti. 6 (aural adj3 vertigo).ab,ti. 7 (labyrinth* adj3 vertigo).ab,ti. 8 (cochlea adj3 hydrops).ab,ti. 9 (vestibular adj3 hydrops).ab,ti. 10 1 or 2 or 3 or 4 or 5 or 6 or 7 or 8 or 9 11 Randomized controlled trial/ 12 Controlled clinical study/ 13 Random$.ti,ab. 14 randomization/ 15 intermethod comparison/ 16 placebo.ti,ab. 17 (compare or compared or comparison).ti. 18 ((evaluated or evaluate or evaluating or assessed or assess) and (compare or compared or comparing or comparison)).ab. 19 (open adj label).ti,ab. 20 ((double or single or doubly or singly) adj (blind or blinded or blindly)).ti,ab. 21 double blind procedure/ 22 parallel group$1.ti,ab. 23 (crossover or cross over).ti,ab. 24 ((assign$ or match or matched or allocation) adj5 (alternate or group$1 or intervention$1 or patient$1 or subject$1 or participant$1)).ti,ab. 25 (assigned or allocated).ti,ab. 26 (controlled adj7 (study or design or trial)).ti,ab. 27 (volunteer or volunteers).ti,ab. 28 human experiment/ 29 trial.ti. 30 11 or 12 or 13 or 14 or 15 or 16 or 17 or 18 or 19 or 20 or 21 or 22 or 23 or 24 or 25 or 26 or 27 or 28 or 29 31 (random$ adj sampl$ adj7 ("cross section$" or questionnaire$1 or survey$ or database$1)).ti,ab. 32 comparative study/ or controlled study/ 33 randomi?ed controlled.ti,ab. 34 randomly assigned.ti,ab. 35 32 or 33 or 34 36 31 not 35 37 Cross‐sectional study/ 38 randomized controlled trial/ or controlled clinical study/ or controlled study/ 39 (randomi?ed controlled or control group$1).ti,ab. 40 38 or 39 41 37 not 40 42 (((case adj control$) and random$) not randomi?ed controlled).ti,ab. 43 (Systematic review not (trial or study)).ti. 44 (nonrandom$ not random$).ti,ab. 45 Random field$.ti,ab. 46 (random cluster adj3 sampl$).ti,ab. 47 (review.ab. and review.pt.) not trial.ti. 48 we searched.ab. 49 review.ti. or review.pt. 50 48 and 49 51 update review.ab. 52 (databases adj4 searched).ab. 53 (rat or rats or mouse or mice or swine or porcine or murine or sheep or lambs or pigs or piglets or rabbit or rabbits or cat or cats or dog or dogs or cattle or bovine or monkey or monkeys or trout or marmoset$1).ti. and animal experiment/ 54 36 or 41 or 42 or 43 or 44 or 45 or 46 or 47 or 50 or 51 or 52 55 30 not 54 56 10 and 55	# 12 #11 AND #10 Indexes=SCI‐EXPANDED, SSCI, A&HCI, CPCI‐S, CPCI‐SSH, BKCI‐S, BKCI‐SSH, ESCI, CCR‐EXPANDED, IC Timespan=All years # 11 TOPIC: ((randomised OR randomized OR randomisation OR randomisation OR placebo* OR (random* AND (allocat* OR assign*) ) OR (blind* AND (single OR double OR treble OR triple) ))) Indexes=SCI‐EXPANDED, SSCI, A&HCI, CPCI‐S, CPCI‐SSH, BKCI‐S, BKCI‐SSH, ESCI, CCR‐EXPANDED, IC Timespan=All years # 10 #9 OR #8 OR #7 OR #6 OR #5 OR #4 OR #3 OR #2 OR #1 Indexes=SCI‐EXPANDED, SSCI, A&HCI, CPCI‐S, CPCI‐SSH, BKCI‐S, BKCI‐SSH, ESCI, CCR‐EXPANDED, IC Timespan=All years # 9 TOPIC: (vestibular NEAR/3 hydrops) Indexes=SCI‐EXPANDED, SSCI, A&HCI, CPCI‐S, CPCI‐SSH, BKCI‐S, BKCI‐SSH, ESCI, CCR‐EXPANDED, IC Timespan=All years # 8 TOPIC: (cochlea NEAR/3 hydrops) Indexes=SCI‐EXPANDED, SSCI, A&HCI, CPCI‐S, CPCI‐SSH, BKCI‐S, BKCI‐SSH, ESCI, CCR‐EXPANDED, IC Timespan=All years # 7 TOPIC: (labyrinth* NEAR/3 vertigo) Indexes=SCI‐EXPANDED, SSCI, A&HCI, CPCI‐S, CPCI‐SSH, BKCI‐S, BKCI‐SSH, ESCI, CCR‐EXPANDED, IC Timespan=All years # 6 TOPIC: (labyrinth* adj3 vertigo) Indexes=SCI‐EXPANDED, SSCI, A&HCI, CPCI‐S, CPCI‐SSH, BKCI‐S, BKCI‐SSH, ESCI, CCR‐EXPANDED, IC Timespan=All years # 5 TOPIC: (aural NEAR/3 vertigo) Indexes=SCI‐EXPANDED, SSCI, A&HCI, CPCI‐S, CPCI‐SSH, BKCI‐S, BKCI‐SSH, ESCI, CCR‐EXPANDED, IC Timespan=All years # 4 TOPIC: (labyrinth* NEAR/3 syndrome) Indexes=SCI‐EXPANDED, SSCI, A&HCI, CPCI‐S, CPCI‐SSH, BKCI‐S, BKCI‐SSH, ESCI, CCR‐EXPANDED, IC Timespan=All years # 3 TOPIC: (labyrinth* NEAR/3 hydrops) Indexes=SCI‐EXPANDED, SSCI, A&HCI, CPCI‐S, CPCI‐SSH, BKCI‐S, BKCI‐SSH, ESCI, CCR‐EXPANDED, IC Timespan=All years # 2 TOPIC: (endolymphatic NEAR/3 hydrops) Indexes=SCI‐EXPANDED, SSCI, A&HCI, CPCI‐S, CPCI‐SSH, BKCI‐S, BKCI‐SSH, ESCI, CCR‐EXPANDED, IC Timespan=All years # 1 TOPIC: (meniere*) Indexes=SCI‐EXPANDED, SSCI, A&HCI, CPCI‐S, CPCI‐SSH, BKCI‐S, BKCI‐SSH, ESCI, CCR‐EXPANDED, IC Timespan=All years	Clinicaltrials.gov menieres or meniere or meniere's | Interventional Studies ICTRP Meniere*

The date restrictions applied to the September 2022 update searches were as follows:

**Table:**

** **	**September 2022 update**
**CENTRAL**	15/09/2021_TO_14/09/2022:CRSINCENTRAL AND CENTRAL:TARGET
**ENT register**	No new records added to register since search was run
**Medline**	23 limit 22 to ed=20210915‐20220914 24 limit 22 to dt=20210915‐20220914 25 23 or 24
**Embase**	57 limit 56 to dd=20210915‐20220914
**Web of Science**	Timespan: 2021‐09‐15 to 2022‐09‐14 (Index Date)
**Clinicaltrials.gov**	First posted from 09/15/2021 to 09/14/2022
**ICTRP**	Date of registration after 15/09/2021
**Google Scholar**	Year: 2021 or 2022

### Appendix 3. Trustworthiness Screening Tool

This screening tool has been developed by Cochrane Pregnancy and Childbirth. It includes a set of predefined criteria to select studies that, based on available information, are deemed to be sufficiently trustworthy to be included in the analysis. These criteria are:

**Research governance**

- Are there any retraction notices or expressions of concern listed on the Retraction Watch Database relating to this study?
- Was the study prospectively registered (for those studies published after 2010)? If not, was there a plausible reason?
- When requested, did the trial authors provide/share the protocol and/or ethics approval letter?
- Did the trial authors engage in communication with the Cochrane Review authors within the agreed timelines?
- Did the trial authors provide IPD data upon request? If not, was there a plausible reason?

**Baseline characteristics**

- Is the study free from characteristics of the study participants that appear too similar (e.g. distribution of the mean (SD) excessively narrow or excessively wide, as noted by Carlisle 2017)?

**Feasibility**

- Is the study free from characteristics that could be implausible? (e.g. large numbers of women with a rare condition (such as severe cholestasis in pregnancy) recruited within 12 months);
- In cases with (close to) zero losses to follow‐up, is there a plausible explanation?

**Results**

- Is the study free from results that could be implausible? (e.g. massive risk reduction for main outcomes with small sample size)?
- Do the numbers randomised to each group suggest that adequate randomisation methods were used (e.g. is the study free from issues such as unexpectedly even numbers of women 'randomised’ including a mismatch between the numbers and the methods, if the authors say 'no blocking was used’ but still end up with equal numbers, or if the authors say they used 'blocks of 4’ but the final numbers differ by 6)?

Studies assessed as being potentially 'high risk’ will be not be included in the review. Where a study is classified as 'high risk’ for one or more of the above criteria we will attempt to contact the study authors to address any possible lack of information/concerns. If adequate information remains unavailable, the study will remain in 'awaiting classification’ and the reasons and communications with the author (or lack of) described in detail.

The process is described in full in Figure 2. 

## Data and analyses

**Comparison 1:** Intratympanic gentamicin versus no treatment/placebo

Outcome or subgroup title	No. of studies	No. of participants	Statistical method	Effect size
1.1 Improvement in vertigo frequency	2		Risk Ratio (M‐H, Random, 95% CI)	Subtotals only
1.1.1 6 to ≤ 12 months	1	32	Risk Ratio (M‐H, Random, 95% CI)	33.00 [2.15, 507.00]
1.1.2 > 12 months	1	22	Risk Ratio (M‐H, Random, 95% CI)	1.63 [0.98, 2.69]
1.2 Improvement in vertigo frequency: sensitivity analysis for complete/substantial improvement	2		Risk Ratio (M‐H, Random, 95% CI)	Subtotals only
1.2.1 6 to ≤ 12 months	1	32	Risk Ratio (M‐H, Random, 95% CI)	33.00 [2.15, 507.00]
1.2.2 > 12 months	1	22	Risk Ratio (M‐H, Random, 95% CI)	7.05 [1.59, 31.32]
1.3 Change in vertigo (global score)	1		Mean Difference (IV, Random, 95% CI)	Subtotals only
1.3.1 6 to ≤ 12 months	1	26	Mean Difference (IV, Random, 95% CI)	‐1.00 [‐1.68, ‐0.32]
1.3.2 > 12 months	1	26	Mean Difference (IV, Random, 95% CI)	‐1.80 [‐2.49, ‐1.11]
1.4 Change in vertigo (frequency) > 12 months	1	22	Mean Difference (IV, Random, 95% CI)	Not estimable
1.5 Change in hearing at > 12 months	2	49	Mean Difference (IV, Random, 95% CI)	3.70 [‐8.29, 15.69]

## Characteristics of studies

### Characteristics of included studies [ordered by study ID]

**Table:** Bremer 2014

***Study characteristics***		
Methods	Parallel‐group, double‐blind RCT with 4 weeks of treatment and intended follow‐up for 2 years Trial prematurely ended due to: slow enrolment; safety concerns due to hearing loss in 1 participant; new publications reporting benefits of intratympanic gentamicin.	
Participants	**Setting: ** 2 hospital clinics at different sites in the Netherlands; recruitment from June 2009 and January 2012 **Sample size:** **Number randomised:** 15 participants **Number completed:** 12 participants **Participant baseline characteristics** **Age: ** Low‐dose gentamicin group: 72.6 years (SD 5 years) High‐dose gentamicin group: 64.5 years (SD 8 years) Control group: 57.3 years (SD 16.7 years) **Gender: ** Low‐dose gentamicin group: 4 male (100%) High‐dose gentamicin group: 3 male (60%): 2 female (40%) Control group: 1 male (20%): 4 female (80%) **Probable/definite Ménière's disease: ** All definite disease (inclusion criterion) **Duration of disease: ** Low‐dose gentamicin group: median 3.3 years (range 0.7 to 7.5 years) High‐dose gentamicin group: median 3.1 years (range 1.1 to 19.6 years) Control group: median 2.5 years (range 0.1 to 18.2 years) **Attack frequency at baseline: ** Not reported **Hearing loss at baseline:** Low‐dose gentamicin group, averaged pure tone hearing loss: Right ear 29.7 dB HL (SD 7.4) Left ear 61.3 dB HL (SD 30.7) High‐dose gentamicin group, averaged pure tone hearing loss: Right ear 59.0 dB HL (SD 8.6) Left ear 46.8 dB HL (SD 21.3) Control group, averaged pure tone hearing loss: Right ear 35.0 dB HL (SD 24.5) Left ear 43.8 dB HL (SD 21.6) **Measure of tinnitus at baseline: ** Not reported **Number of participants with bilateral disease: ** None; inclusion criterion of unilateral disease **Inclusion criteria:** Unilateral Ménière's disease, according to AAO‐HNS 1995 criteria. Resistance to conservative medical treatments for at least 6 months (type of treatment not specified). DHI score of at least 30 points. Written, informed consent. Compromised hearing on the affected side without fluctuations. **Exclusion criteria:** Ipsilateral middle ear pathology. Contralateral ear pathology or contralateral hearing loss. Allergy to aminoglycosides. Previous treatment with intratympanic gentamicin. Causes other than Ménière's disease (assessed with a diagnostic protocol that included vestibular tests, MRI of the cerebellopontine angle, clinical history and physical examination). **Diagnostic criteria for Ménière's disease** Definite Ménière's disease, according to the AAO‐HNS 1995 criteria	
Interventions	**Intervention (n = 10 randomised, n = 8 completed)** Intratympanic gentamicin. Two treatment arms (pooled for the purposes of this review). Received 2 injections of gentamicin and 2 injections of placebo (0.9% saline) or 4 injections of gentamicin. One injection was administered per week over a period of 4 weeks. Placebo/gentamicin was given in a random order. Concentration of gentamicin 40 mg/mL, quantity instilled is not reported. Methods of administration not stated ‐ presumed to be direct injection as no mention of ventilation tubes. **Comparator (n = 5 randomised, n = 4 completed)** Placebo; received intratympanic injection of normal saline, once per week for 4 weeks **Background interventions administered to all participants** Quote from article: "After ITG [intratympanic gentamicin] treatment patients were advised to visit the physiotherapist for, for example, Cawthorn‐Cooksey exercises or other patient‐specific exercises". It is not clear whether these exercises were only offered to those in the gentamicin group, or were also offered to those in the placebo group.	
Outcomes	**Primary outcomes relevant to this review:** **Improvement in vertigo** Not reported **Change in vertigo** Not reported **Serious adverse events** Article states: "Harms were reported following the CONSORT extension for harms." No events are reported, except for hearing outcomes. **Secondary outcomes relevant to this review:** **Disease‐specific health‐related quality of life** Assessed with the DHI **Hearing** Assessed with pure tone audiogram at 0.5 kHz, 1 kHz, 2 kHz and 4 kHz; reported as change in hearing threshold **Tinnitus ** Not reported **Other adverse effects** Article states: "Harms were reported following the CONSORT extension for harms." No events are reported, except for hearing outcomes. **Other outcomes reported in the study:** According to the trial protocol, the following were planned to be assessed, but are not reported: Activities balance confidence scale (ABC) Control of vertigo Tinnitus Handicap Inventory (THI) Electronystagmography Rescue medication	
Funding sources	Quote: "There was no source of funding for this study"	
Declarations of interest	Quote: "The authors declare that they have no competing interests"	
Notes	**Research integrity checklist:** No retractions/expressions of concern were identified Study protocol was registered prospectively EudraCT number 2006‐005913‐37. Authors also supplied a copy of the study protocol. Plausible loss to follow‐up was reported No implausible results were detected No indication of inadequate randomisation, given the small number of participants recruited	
***Risk of bias***		
**Bias**	**Authors' judgement**	**Support for judgement**
Random sequence generation (selection bias)	Low risk	Quote: "For assignment of the participants a computer‐generated list of random numbers was used". Comment: adequate randomisation method.
Allocation concealment (selection bias)	Unclear risk	Quote: "For assignment of the participants a computer‐generated list of random numbers was used". Comment: no details regarding concealment of the list of random numbers.
Blinding of participants and personnel (performance bias) All outcomes	Unclear risk	Quote: From trial protocol: "Neither the investigator, nor the treating physician, nor the subject is aware of the sequence for randomization." "After ITG treatment patients were advised to visit the physiotherapist for, for example, Cawthorn‐Cooksey exercises or other patient‐specific exercises." Comment: although the intention was for the trial to be double‐blind, there is a suggestion in the article that participants in the intratympanic gentamicin groups had access to a physiotherapist if required, and it is not clear if this was also offered to the placebo group. Therefore, there is the potential for blinding to have been compromised.
Blinding of outcome assessment (detection bias) All outcomes	Unclear risk	Quote: From trial protocol: "Neither the investigator, nor the treating physician, nor the subject is aware of the sequence for randomization." "After ITG treatment patients were advised to visit the physiotherapist for, for example, Cawthorn‐Cooksey exercises or other patient‐specific exercises." Comment: although the intention was for the trial to be double‐blind, there is a suggestion in the article that participants in the intratympanic gentamicin groups had access to a physiotherapist if required, and it is not clear if this was also offered to the placebo group. Therefore, there is the potential for blinding to have been compromised. Primary outcome was reported by participants themselves.
Incomplete outcome data (attrition bias) All outcomes	High risk	Comment: few participants and unequal dropout between groups, with loss to follow‐up predominantly in the low‐dose gentamicin group (2/5 participants). This is likely to have influenced the results. One patient (group 2) withdrew from the study after randomisation because she did not want placebo. This reason for withdrawal is unlikely to be related to the true outcomes (DHI score and hearing change). Another patient (group not reported) withdrew from the study after 2 injections because he suffered from Tumarkins crises. This reason for withdrawal is likely to be related to the true outcome for DHI score. One patient (group 2) died during the study as a result of co‐morbidity. This is unlikely to be related to the true outcomes (DHI score and hearing change). Therefore, for 1 of the 3 losses to follow‐up, the reason for withdrawal was likely to be related to the putative true outcome. Furthermore, given the small number of participants, 2 losses from group 2 (40%) is likely to exert a differential potential for bias across groups.
Selective reporting (reporting bias)	High risk	Comment: protocol indicates that a number of additional outcomes will be assessed, but these are not reported, including control of vertigo, ABC scale, THI, electronystagmography and rescue medication.
Other bias	Low risk	Comment: no other potential source of bias was detected.

**Table:** Choudhary 2019

***Study characteristics***		
Methods	Quasi‐randomised, non‐blinded, parallel‐group, controlled trial of up to 9 weeks of treatment and further 4 months of follow‐up (6 months duration in total)	
Participants	**Setting: ** Single‐centre study, conducted in the ENT department of a tertiary referral centre in India **Sample size:** **Number randomised:** 32 participants **Number completed:** 32 participants **Participant baseline characteristics** **Age: ** Not reported **Gender: ** Gentamicin group: 8 male (50%): 8 female (50%) Control group: not reported **Probable/definite Ménière's disease: ** Not reported **Duration of disease:** Not reported **Attack frequency at baseline:** Not reported **Hearing loss at baseline:** Not reported **Measure of tinnitus at baseline: ** Not reported **Number of participants with bilateral disease: ** Not reported **Inclusion criteria:** Ménière's disease patients with normal middle ear function, without systemic diseases like diabetes mellitus, bleeding disorders etc., who had never been given intratympanic gentamicin before and failed to respond to 1‐year medical therapy (type of therapy not stated) **Exclusion criteria:** Other causes of vertigo, sensorineural hearing loss, tinnitus and aural fulness; impaired middle ear function; systemic diseases like diabetes mellitus, bleeding disorders etc; previous treatment with intratympanic gentamicin; or responded to 1‐year medical therapy **Diagnostic criteria for Ménière's disease:** Definite Ménière's disease, according to the AAO‐HNS 1995	
Interventions	**Intervention (n = 16 randomised, n = 16 completed)** Intratympanic gentamicin. Between 1 and 3 injections of gentamicin (1 mL of a 20 mg/mL solution) were administered through the tympanic membrane with a tuberculin syringe fitted with a small spinal needle. The solution was left in the middle ear for 30 minutes with the patient lying flat and not swallowing. An interval of at least 3 weeks was left between injections. The 2nd injection was only given if the participant had experienced a vertigo episode in the intervening 2 weeks (presumably this was also the case for the 3rd injection). **Comparator (n = 16 randomised, n = 16 completed)** Conservative treatment: "not given intratympanic gentamicin". Presumed to be no treatment. **Background interventions administered to all participants** None reported.	
Outcomes	**Primary outcomes relevant to this review:** **Improvement in vertigo** Assessed using the AAO‐HNS 1995 criteria for complete resolution, substantial control or limited control at 6 months after treatment **Change in vertigo** Not reported **Serious adverse events** Not apparently systematically assessed; some information on progression of hearing loss only **Secondary outcomes relevant to this review:** **Disease‐specific health‐related quality of life** Not reported **Hearing** No continuous data reported; some information on progression of hearing loss, but it is unclear how this was assessed **Tinnitus ** Not reported **Other adverse effects** Not reported **Other outcomes reported in the study:** No further outcomes reported	
Funding sources	Not reported	
Declarations of interest	Article states: "Financial or Other Competing Interest: None"	
Notes	**Research integrity checklist:** No retractions/expressions of concern noted No trial registration or protocol was identified Baseline characteristics of the groups are not reported, therefore unable to assess whether there are excessive similarities No loss to follow‐up was reported All participants in the intervention group experienced substantial or complete control of vertigo (14/16 (87.5%) experiencing complete control). All participants in the control group experienced either worse control (7/16 (43.8%)) or needed secondary treatment due to disability from vertigo (9/16 (56.3%)). While this is plausible given the small sample, the risk of worsening control is extremely high in the control group in comparison to the intervention group. 16 participants were recruited to each group through quasi‐randomisation. True randomisation was conducted initially using a random number table. However, if those allocated to the intervention group did not give subsequent consent, they were added to the control group. The "next" participant was then placed in the intervention group, presumably to achieve a balance in numbers across groups.	
***Risk of bias***		
**Bias**	**Authors' judgement**	**Support for judgement**
Random sequence generation (selection bias)	High risk	Quote: "We numbered the patients who qualified for our study depending upon the inclusion criteria & then used random number table to assign patients into intervention group & control group". "Patients were free to consent for the intervention & if any one did not give consent, he/she was put into the control group & the next patient was considered for placement in intervention group." Comment: true randomisation was conducted initially using a random number table. However, if those allocated to the intervention group did not give subsequent consent, they were added to the control group. The "next" participant was then placed in the intervention group, presumably to achieve a balance in numbers across groups. This could be considered quasi‐randomisation. There is considerable risk that a balance of important prognostic factors (especially those that might be associated with a preference not to provide consent) may not have been achieved across groups. No baseline characteristics were provided for each group, making it difficult to judge whether such balance had been achieved.
Allocation concealment (selection bias)	High risk	Quote: "Patients were free to consent for the intervention & if any one did not give consent, he/she was put into the control group & the next patient was considered for placement in intervention group." Comment: allocation was not concealed, and participant could choose to change groups if they required.
Blinding of participants and personnel (performance bias) All outcomes	High risk	Comment: no blinding of participants (allocation was to an active intervention or no treatment).
Blinding of outcome assessment (detection bias) All outcomes	High risk	Comment: outcome measures were reported by unblinded participants. The measurement of all outcomes could have been influenced by knowledge of group assignation.
Incomplete outcome data (attrition bias) All outcomes	Low risk	Comment: complete follow‐up is reported with no attrition.
Selective reporting (reporting bias)	High risk	Comment: no protocol was available for comparison. However, information for some outcomes was reported incompletely and for only one intervention arm. A specified aim of the study was to compare groups for the degree of sensorineural hearing loss. However, the degree of hearing loss was not described and the outcome was presented dichotomised into "further hearing loss" and "no further hearing loss".
Other bias	Unclear risk	Comment: limited details are presented regarding the study methods. It is unclear how participants recorded their vertigo episodes during the follow‐up period. Methods of assessment for hearing loss were not described.

**Table:** Postema 2008

***Study characteristics***		
Methods	Parallel‐group, double‐blind RCT with 4 weeks of treatment and further 12 months of follow‐up	
Participants	**Setting: ** Outpatient clinical setting in a tertiary neuro‐otologic centre in the Netherlands; trial study dates were not reported **Sample size:** **Number randomised:** 28 participants **Number completed:** 26 participants **Participant baseline characteristics** **Age: ** Gentamicin group: median 55 years Placebo group: median 53 years **Gender: ** Not reported **Probable/definite Ménière's disease: ** All definite disease (inclusion criterion) **Duration of disease: ** Not reported **Attack frequency at baseline: ** Not reported **Hearing loss at baseline:** Gentamicin group: mean hearing loss for affected ear 56 dB Placebo group: mean hearing loss for affected ear 53 dB **Measure of tinnitus at baseline: ** Not reported **Number of participants with bilateral disease: ** None; bilateral disease was an exclusion criterion **Inclusion criteria:** Diagnosed as having Ménière's disease according to the AAO‐HNS 1995 criteria for definite Ménière's disease. The patient’s most annoying complaint had to be vertigo, the vestibulum had to show a caloric response and conservative medical treatment with betahistine was unsuccessful. **Exclusion criteria:** Causes other than Ménière's disease (excluded using a diagnostic protocol including vestibular tests, MRI of cerebellopontine angle, clinical history and physical examination). Bilateral disease. The ear to be treated was the best hearing ear. **Diagnostic criteria for Ménière's disease** AAO‐HNS 1995 for definite Ménière's disease	
Interventions	**Intervention (n = 16 randomised, n = 16 completed)** Gentamicin group: 0.4 mL (30 mg/mL) gentamicin sulphate (total 12 mg) was injected once a week through the ventilation tube with a small needle. This occurred on 4 occasions, each separated by a week. **Comparator (n = 12 randomised, n = 10 completed)** Placebo group. The same procedure was used (as above) but with 0.4 mL placebo solution. The composition of this was not reported. **Background interventions administered to all participants** All participants had a ventilation tube inserted 4 weeks before the start of their therapy	
Outcomes	**Primary outcomes relevant to this review:** **Improvement in vertigo** Not reported **Change in vertigo** Global change in severity, assessed with a 4‐point, self‐reported scale (0 = none, 1 = mild, 2 = moderate, 3 = severe). We assume that this would encompass all aspects of vertigo frequency, duration and intensity, but this is not clear. **Serious adverse events** Not reported **Secondary outcomes relevant to this review:** **Disease‐specific health‐related quality of life** Not reported **Hearing** Assessed with the change from baseline in the extended Fletcher index (average of losses at 0.5 kHz, 1 kHz, 2 kHz and 4 kHz with pure tone audiogram) **Tinnitus ** No validated scale was used to assess tinnitus impact; tinnitus severity was assessed with a 4‐point scale (0 = none, 1 = mild, 2 = moderate, 3 = severe) **Other adverse effects** Not reported **Other outcomes reported in the study:** Aural fullness Association of vertigo and aural fullness scores	
Funding sources	Not reported	
Declarations of interest	Not reported	
Notes	**Research integrity checklist:** No retractions/expressions of concern were identified Trial registration was not applicable as this study was published prior to 2010 We were unable to fully assess baseline characteristics of the groups due to the limited data presented Close to zero participants were lost to follow‐up, but this may be expected given the small number recruited No implausible results were identified There was no concern regarding inadequate randomisation	
***Risk of bias***		
**Bias**	**Authors' judgement**	**Support for judgement**
Random sequence generation (selection bias)	Unclear risk	Quote: "prospective, double‐blind, randomized, placebo‐controlled clinical trial". Comment: no information on methods of randomisation are reported.
Allocation concealment (selection bias)	Unclear risk	Quote: "During the final visit the double‐blind code was broken. Before that time this code was only known to one of the hospital pharmacists". Comment: no details on how the code was secured.
Blinding of participants and personnel (performance bias) All outcomes	Low risk	Quote: "During the final visit the double‐blind code was broken. Before that time this code was only known to one of the hospital pharmacists." Comment: lack of detail, but study is described as double‐blind, and it is likely that participants would have been unable to tell if they were receiving active treatment or placebo. Article indicates that study personnel were blinded (see quote above).
Blinding of outcome assessment (detection bias) All outcomes	High risk	Quote: "During the final visit the double‐blind code was broken. Before that time this code was only known to one of the hospital pharmacists." Comment: the nature of the outcomes was such that measurement could be subject to bias given knowledge of the assigned group. Blinding was described as 'double' but few further details were provided. Blinding was 'broken' at 12 months post‐treatment. This was stated to be during the final visit, suggesting that participants and personnel were made aware of group allocation during this visit.
Incomplete outcome data (attrition bias) All outcomes	Low risk	Comment: dropout is comparatively low (2/28 participants), although both dropouts were in the placebo group, and no information was provided regarding the cause for dropout. Dropout is probably not large enough to have a meaningful impact on the estimated effect size.
Selective reporting (reporting bias)	Unclear risk	Comment: no protocol was available to assess whether all pre‐specified outcomes were reported.
Other bias	High risk	Comment: unvalidated scales used for all outcome measures (except hearing). Unclear if participants assessed their symptoms over a given period of time, or simply on the day of their appointment ‐ the authors do not report the use of a symptom diary.

**Table:** Stokroos 2004

***Study characteristics***		
Methods	Parallel‐group, double‐blind RCT with interventions every 6 weeks until symptoms were controlled, and follow‐up for at least 6 months (up to 28 months in total)	
Participants	**Setting: ** Tertiary referral neurotologic centre in the Netherlands; study recruitment was from October 2000 until October 2002 **Sample size:** **Number randomised:** 22 participants **Number completed:** 22 participants. No dropouts were reported, therefore we presume that complete follow‐up was achieved. **Participant baseline characteristics** **Age: ** Gentamicin group: mean 59 years (range 34 to 74) Control group: mean 58 years (range 45 to 70) **Gender: ** Only reported for entire cohort; 13 males (59%): 9 females (41%) **Probable/definite Ménière's disease: ** All definite disease (inclusion criterion) **Duration of disease: ** Not reported **Attack frequency at baseline: ** Gentamicin group: mean 74 attacks per year (SD 114) Control group: mean 25 attacks per year (SD 31) **Hearing loss at baseline:** Gentamicin group: mean 60dB HL (SD 18.7) Control group: mean 53 dB HL (SD 16.5) **Measure of tinnitus at baseline: ** Not reported **Number of participants with bilateral disease: ** None; inclusion criterion of unilateral disease **Inclusion criteria:** Definite, active Ménière's disease, according to AAO‐HNS 1995 criteria. Known underlying cause excluded using a diagnostic protocol (not described). Conservative/medical treatment for at least 6 months was unsuccessful. Incapacitating vertigo attacks occurring at least monthly and recorded for at least 6 months. Unilateral pathology. Informed consent obtained. **Exclusion criteria:** Contralateral neuro‐otological pathology. Ipsilateral middle ear pathology. Allergy to aminoglycosides. Additional exclusion criteria were applied throughout the study: Cumulative gentamicin dose ≥ 360 mg for 12 applications Cumulative treatment time after first treatment > 6 months Perceptive hearing loss after treatment ≥ 15 dB for 2 subsequent octave steps in the pure tone audiogram **Diagnostic criteria for Ménière's disease** AAO‐HNS 1995 criteria for definite disease	
Interventions	**Intervention (n = 12 randomised, n = 12 completed)** Gentamicin group: 4 mL of 30 mg/mL gentamicin in a buffer solution (pH6.4) was injected via a spinal needle after local anaesthesia with lidocaine spray. "After aspirating any remaining lidocaine, a paracentesis is performed just anterior to the umbo [...] The hypo‐ and mesotympanum are filled with either gentamicin or placebo until the fluid meniscus is touching the paracentesis opening and fluid flows back into the external meatus". The patient remained supine with the affected ear upwards for 45 minutes. Applications were repeated every 6 weeks until either control of symptoms was achieved, or one of the exclusion criteria was met: Cumulative gentamicin dose ≥ 360 mg for 12 applications Cumulative treatment time after first treatment > 6 months Perceptive hearing loss after treatment ≥ 15 dB for 2 subsequent octave steps in the pure tone audiogram The mean number of applications performed was 1.5 (SD 0.51) **Comparator (n = 10 randomised, n = 10 completed)** Placebo group: the procedure was identical to that described above, but only the buffer solution was used. The mean number of applications performed was 2.8 (SD 2.7). **Background interventions administered to all participants** None reported. It is unclear whether baseline conservative/medical treatment was continued through the trial.	
Outcomes	**Primary outcomes relevant to this review:** **Improvement in vertigo** Assessed based on patient report of improvement. The authors state that "the number of vertiginous spells were noted", although there is very little information regarding how participants assessed their symptoms. The outcome is reported simply as "no complaints", "significant reduction", "some reduction" and "no benefit". It is unclear if this was systematically assessed in the same way for each group or relied only on the patient style of reporting. Reported at final follow‐up (range 6 to 28 months). **Change in vertigo** Assessed as the change in frequency of vertigo at final follow‐up (range 6 to 28 months) **Serious adverse events** Not reported **Secondary outcomes relevant to this review:** **Disease‐specific health‐related quality of life** Not reported **Hearing** Reported as the hearing loss according to pure tone average at 0.5 kHz, 1 kHz, 2 kHz and 4 kHz at final follow‐up (range 6 to 28 months) **Tinnitus ** Not reported **Other adverse effects** Not reported **Other outcomes reported in the study:** Vestibular symmetry Caloric response	
Funding sources	No details provided	
Declarations of interest	No details provided	
Notes	**Research integrity checklist:** No retractions/expressions of concern were identified No trial registration was necessary as this trial was published before 2010 Limited data on the baseline characteristics of participants was reported. Baseline vertigo attacks were much more frequent in the gentamicin group than placebo, which may indicate an issue with randomisation. No loss to follow‐up was reported, and no reasons are given for this No concerns over the adequacy of randomisation	
***Risk of bias***		
**Bias**	**Authors' judgement**	**Support for judgement**
Random sequence generation (selection bias)	Unclear risk	Quote: "Randomization was performed by the hospital pharmacist, who was the only person who knew whether placebo or gentamicin was given before the end of the study period". Comment: insufficient information was given about sequence generation.
Allocation concealment (selection bias)	Unclear risk	Quote: "Randomization was performed by the hospital pharmacist, who was the only person who knew whether placebo or gentamicin was given before the end of the study period". Comment: no details provided on how allocation was concealed. Concern over baseline imbalance in vertigo frequency, which could indicate that people with worse symptoms were preferentially recruited to the gentamicin group. However, this would potentially bias the effect size towards the null.
Blinding of participants and personnel (performance bias) All outcomes	Low risk	Quote: "Randomization was performed by the hospital pharmacist, who was the only person who knew whether placebo or gentamicin was given before the end of the study period". Comment: all parties involved in the trial except the hospital pharmacist were blind to treatment allocation.
Blinding of outcome assessment (detection bias) All outcomes	Low risk	Quote: "Randomization was performed by the hospital pharmacist, who was the only person who knew whether placebo or gentamicin was given before the end of the study period". Comment: all parties involved in the trial except the hospital pharmacist were blind to treatment allocation. Outcomes were reported by blinded participants.
Incomplete outcome data (attrition bias) All outcomes	Low risk	Comment: no missing data are reported.
Selective reporting (reporting bias)	High risk	Comment: results state that the number of vertiginous attacks was 0 for all participants in the gentamicin group. Discussion states that a recurrence of vertigo attacks occurred in 1 participant 1 to 2 years after successful ablation. No information on timing of outcome assessment. Some outcomes were described in the methods section, but not reported among the results: subjective hearing changes and speech audiometry.
Other bias	High risk	Comment: unclear how participants assessed vertigo frequency. Varying number of interventions in different participants, which may lead to differential period of follow‐up between the 2 groups. No detail is given on the measurement of outcomes in terms of the precise methods, personnel involved, their training and the reliability of measurements.

**Table:** Ul Shamas 2017

***Study characteristics***		
Methods	Parallel‐group RCT. Presumed to be an unblinded study. Single administration of intervention followed by 6 months of follow‐up. Trial involved 3 arms: IT corticosteroids, IT gentamicin, IT placebo. For the purposes of this review only the arms considering IT gentamicin and IT placebo have been reported.	
Participants	**Setting: ** Single‐centre trial conducted at a district hospital in India from February to August 2015 **Sample size: ** **Number randomised:** 40 participants **Number completed:** 40 participants **Participant baseline characteristics** **Age:** Not reported **Gender:** Not reported **Probable/definite Ménière's disease:** Not reported **Duration of disease:** Not reported **Attack frequency at baseline:** Not reported **Hearing loss at baseline:** IT gentamicin group: mean pure tone average hearing threshold at speech frequencies: 50 (SD not reported) Placebo group: mean pure tone average hearing threshold at speech frequencies: 48 (SD not reported) **Measure of tinnitus at baseline:** IT gentamicin group: mean THI 'grade' was 4 at baseline (SD not reported) Placebo group: mean THI 'grade' was 2.5 at baseline (SD not reported) It is unclear what is meant by the THI 'grade' **Number of participants with bilateral disease:** Not reported **Inclusion criteria:** Ménière's disease according to the criteria of the AAO‐HNS 1995. Persistent symptoms despite "maximal medical treatment" (this treatment is not described further in the paper). **Exclusion criteria:** None reported	
Interventions	**Intervention (n = 20 randomised, n = 20 completed)** Gentamicin 2 mL (40 mg/mL) was administered (80 mg total) using a 27 G spinal needle and a 2 mL syringe. The drug was administered in "the anterograde inferior quadrant of the tympanic membrane", with the head tilted to the normal ear and maintained in position for 20 minutes. **Comparator (n = 20 randomised, n = 20 completed)** Intratympanic placebo (composition not stated) was administered in the same way as with the active drug **Background interventions administered to all participants** None reported	
Outcomes	**Primary outcomes relevant to this review:** **Improvement in vertigo** Not reported **Change in vertigo** Not reported **Serious adverse events** Not fully reported; some data on participants with profound hearing loss only **Secondary outcomes relevant to this review:** **Disease‐specific health‐related quality of life** Not reported **Hearing** Not reported in sufficient detail for inclusion in the review. Data are reported for the whole cohort of study participants, not for individual groups, therefore a comparison cannot be made between the groups. **Tinnitus ** THI 'grade' is reported for the individual groups, but the variance is not reported, therefore these data cannot be included in a meta‐analysis **Other adverse effects** Not reported fully ‐ no information on which groups participants were assigned to **Other outcomes reported in the study:** Nature of the tinnitus Hearing change across all cohort, not for individual groups	
Funding sources	Not reported	
Declarations of interest	Not reported	
Notes	**Research integrity checklist:** No retractions/expressions of concern No trial registration or protocol was identified Unable to assess baseline characteristics of the groups, as these are not reported fully Complete follow‐up was reported. No reasons for this were provided. No implausible results were noted for this comparison (IT gentamicin versus placebo/no treatment) Equal numbers of participants were allocated to each group, without mention of blocked randomisation	
***Risk of bias***		
**Bias**	**Authors' judgement**	**Support for judgement**
Random sequence generation (selection bias)	Unclear risk	Quote: "60 consecutive patients of Meniere’s disease were randomly divided into 3 groups of twenty patients each." Comment: very limited information regarding randomisation.
Allocation concealment (selection bias)	Unclear risk	Comment: no details regarding methods used to ensure group allocation was concealed.
Blinding of participants and personnel (performance bias) All outcomes	High risk	Comment: open‐label study. No blinding was used.
Blinding of outcome assessment (detection bias) All outcomes	High risk	Comment: open‐label trial. No blinding of outcome assessors was reported.
Incomplete outcome data (attrition bias) All outcomes	Unclear risk	Comment: no details provided on attrition/exclusions from the study. It is possible that hearing and tinnitus outcome data were either not measured, or not reported for participants who did not have hearing loss or tinnitus at the start of the study: "Patients with hearing loss underwent PTA before treatment and 2 weeks and 6 months post intra tympanic medication." "Patients with tinnitus were graded using tinnitus handicap inventory before and after treatment". (Note that only 52 of the 60 trial participants had tinnitus at the start of the study, and their group assignments were not reported). This could affect interpretation of the rate of profound sensorineural hearing loss among this treated with intratympanic gentamicin.
Selective reporting (reporting bias)	High risk	Comment: no published protocol or trial registration. Study does not apparently assess any vertigo outcomes, which would be considered key for Ménière's disease. Reporting of some outcomes was incomplete ‐ for example, the mean post‐treatment PTA for group B (intratympanic dexamethasone) was not reported; the rate of sensorineural hearing loss was not reported for groups B and C (intratympanic dexamethasone and saline respectively).
Other bias	High risk	Comment: very limited details provided regarding the nature and conduct of the study. Single author RCT with short period of recruitment for 60 participants in a single centre. The training and reliability of personnel assessing outcomes, and the validity of the instruments used, were not reported. No detail was provided on how many participants had unilateral/bilateral disease, and nor was the unit of analysis (person/ear) clarified. There was also a lack of data that could be used to compare outcomes across groups.

dB: decibels; DHI: Dizziness Handicap Inventory; IT: intratympanic; MRI; magnetic resonance imaging; PTA: pure tone average; RCT: randomised controlled trial; SD: standard deviation; THI: Tinnitus Handicap Inventory

### Characteristics of excluded studies [ordered by study ID]

**Table:**

Study	Reason for exclusion
Conde 1965	This is a review article on the treatment of vertigo, not an RCT
Diamond 2003	This is a systematic review; the reference list has been checked to ensure that any relevant trials are included
Dimitriadis 2017	This is a review article; the reference list has been checked to ensure any relevant studies are included
Graybiel 1967	This is not an RCT
Guo 2016	This is not an RCT
Hao 2022	This is a network meta‐analysis, which does not include any new data; the reference list has been checked to ensure that any relevant studies are included
Huon 2012	This is a systematic review; the reference list has been checked to ensure any relevant studies are included
Nedzelski 1992	This is a cohort study, not an RCT
Richards 1971	This is a review article, not an RCT
Syed 2015	This is a systematic review with no new data; the reference list has been checked to ensure any relevant articles are included
Thabet 2008	This is not an RCT

RCT: randomised controlled trial

## Differences between protocol and review

For clarity, we have renamed the review "Intratympanic gentamicin for Ménière's disease" rather than "Intratympanic aminoglycosides for Ménière's disease", as we only identified data regarding gentamicin. 

Due to the paucity of data for most outcomes, we were unable to carry out a number of pre‐planned sensitivity analyses and subgroup analyses. However, we added one unplanned sensitivity analysis in this review. As described in Sensitivity analysis, we assessed whether changing the planned outcome "improvement in vertigo" to "complete resolution or substantial improvement in vertigo" would alter the effect estimates. As this was a post hoc change, the results should be interpreted with caution. However, we considered that people with Ménière's disease may want to know if an intervention had a marked effect on their vertigo symptoms, rather than a more modest change. 

We planned to use the Trustworthiness Screening Tool from Cochrane Pregnancy and Childbirth to identify studies for inclusion in the main analysis. However, as described in Selection of studies, we did not exclude studies from the main analysis on the basis of concerns whilst using this tool. We considered that the overall certainty of the review findings (all very low‐ or low‐certainty) would not be impacted by this decision. 
